# Homonuclear Simplified Preservation of Equivalent Pathways Spectroscopy

**DOI:** 10.1021/acs.jpclett.4c00991

**Published:** 2024-06-10

**Authors:** Evgeny Nimerovsky, Spyridon Kosteletos, Sascha Lange, Stefan Becker, Adam Lange, Loren B. Andreas

**Affiliations:** † Department of NMR-Based Structural Biology, Max Planck Institute for Multidisciplinary Sciences, Am Fassberg 11, Göttingen 37077, Germany; ‡ Department of Molecular Biophysics, Leibniz-Forschungsinstitut für Molekulare Pharmakologie, Robert-Rössle-Straße 10, Berlin 13125, Germany

## Abstract

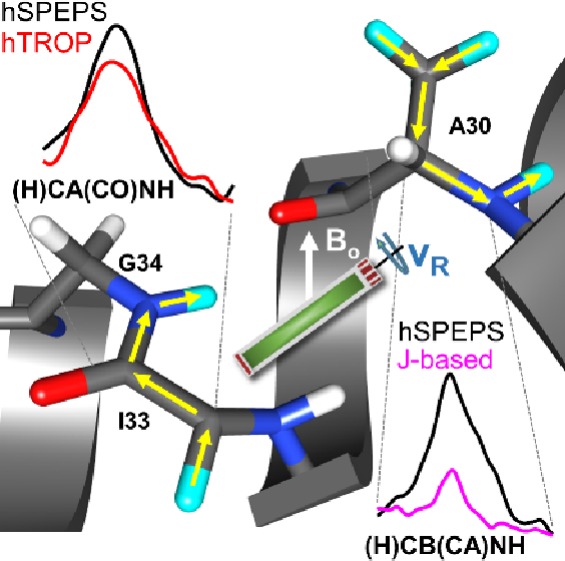

Recently developed homonuclear transverse mixing optimal control pulses (hTROP) revealed an elegant way to enhance the detected signal in multidimensional magic-angle spinning (MAS) nuclear magnetic resonance experiments. Inspired by their work, we present two homonuclear simplified preservation of equivalent pathways spectroscopy (hSPEPS) sequences for recoupling CA–CO and CA–CB dipolar couplings under fast and ultrafast MAS rates, theoretically enabling a √2 improvement in sensitivity for each indirect dimension. The efficiencies of hSPEPS are evaluated for non-deuterated samples of influenza A M2 and bacterial rhomboid protease GlpG under two different external magnetic fields (600 and 1200 MHz) and MAS rates (55 and 100 kHz). Three-dimensional (H)CA(CO)NH, (H)CO(CA)NH, and (H)CB(CA)NH spectra demonstrate the high robustness of hSPEPS elements to excite carbon–carbon correlations, especially in the (H)CB(CA)NH spectrum, where hSPEPS outperforms the *J*-based sequence by a factor of, on average, 2.85.

The efficiency of homonuclear dipolar recoupling elements^1−4^ in transferring the signal to directly bonded spins plays a crucial role in obtaining resonance assignments because the required experiment time decreases with the square of the efficiency. Resonance assignment is typically a time-consuming first step in nuclear magnetic resonance (NMR)-based structural biology,^5−14^ motivating the development of more efficient pulse sequences. Proton-detected experiments typically incorporate carbon–carbon transfer among backbone resonances in the construction of multidimensional magic-angle spinning (MAS) NMR experiments^15−20^ for amino acid assignment in proteins.^21−26^ Notably, carbon–carbon transfer appears in the more challenging experiments that are based on amide proton detection.^27^ The most commonly encountered homonuclear transfers needed for protein assignment are CA–CO transfer, which propagates the signal down the protein backbone, and CA–CB transfer, which is used to record the rich information regarding residue type that is inherent in the CB chemical shift.

For solid protein samples, homonuclear carbon transfer can be achieved via either dipolar coupling (∼2 kHz) or *J* coupling (32–55 Hz). Theoretically, *J*-coupling-based transfer can provide 100% transfer efficiency^28−34^ and becomes competitive for samples exhibiting long relaxation times. On the other hand, transfer based on the stronger dipolar coupling can be accomplished in a shorter time and is logically competitive when relaxation becomes a limiting factor.^28,35^ Theoretically, some dipolar recoupling elements also reach 100% transfer efficiency for an isolated spin pair,^36^ and a suite of proton-detected assignment experiments has been developed around dipolar transfer as well.^26,37^

While it is difficult to beat the exceptional efficiency observed using *J*-based transfers for microcrystalline proteins,^26,27,38^ our interest in membrane proteins that exhibit shorter transverse relaxation times of only 15–20 ms motivated further method development. Recent reports detailed the use of optimal control to find sequences that can transfer both *x* and *y* components of magnetization and, therefore, theoretically improve sensitivity by up to √2.^39,40^ The resulting sequences, known as transverse mixing optimal control pulses (TROP) adapt the preservation of equivalent pathways (PEP) framework for MAS NMR. We previously developed simplified preservation of equivalent pathways spectroscopy (SPEPS)^41^ for heteronuclear transfer.

Here, we describe two novel double-quantum dipolar recoupling pulse sequences, of which one is optimized for large chemical shift differences and the other is optimized for relatively small chemical shift differences. These are demonstrated here for CA–CB and CA–CO recoupling, respectively, and are dubbed homonuclear simplified preservation of equivalent pathways spectroscopy (hSPEPS). hSPEPS^CA–CO^ and hSPEPS^CA–CB^ require short mixing times of around 1 ms. hSPEPS^CA–CO^ is used for CA to CO or CO to CA transfer, and hSPEPS^CA–CB^ is efficient in general for aliphatic–aliphatic transfer. These sequences simultaneously transfer both transverse components of magnetization, which allows √2 improvement in sensitivity for each indirect dimension.^39−42^

Homonuclear simplified preservation of equivalent pathways spectroscopy sequences are amplitude- and phase-modulated sequences,^43−45^ in which two amplitudes and one phase are applied per rotor period. The hSPEPS^CA–CB^ (Figure 1A) and hSPEPS ^CA–CO^ (Figure 1B) pulse sequences are constructed from 16 or 32 rotor periods, respectively. The sequences are repeated as necessary to reach the desired mixing time. In each rotor period, an amplitude-modulated pulse is applied whose phase is based on *XY* phase cycling (Figure 1).^46^ The amplitude modulation is symmetric within a rotor period with two different amplitudes, as shown in panels A and B of Figure 1. For hSPEPS^CA–CB^, the total flip angle of the single amplitude-modulated pulse is 180°. For hSPEPS^CA–CO^, the total flip angle is approximately 165.5°, without considering offset effects, with the optimal radio frequency (rf)-field power varied slightly depending upon the MAS rate and spectrometer frequency.

**Figure 1:**
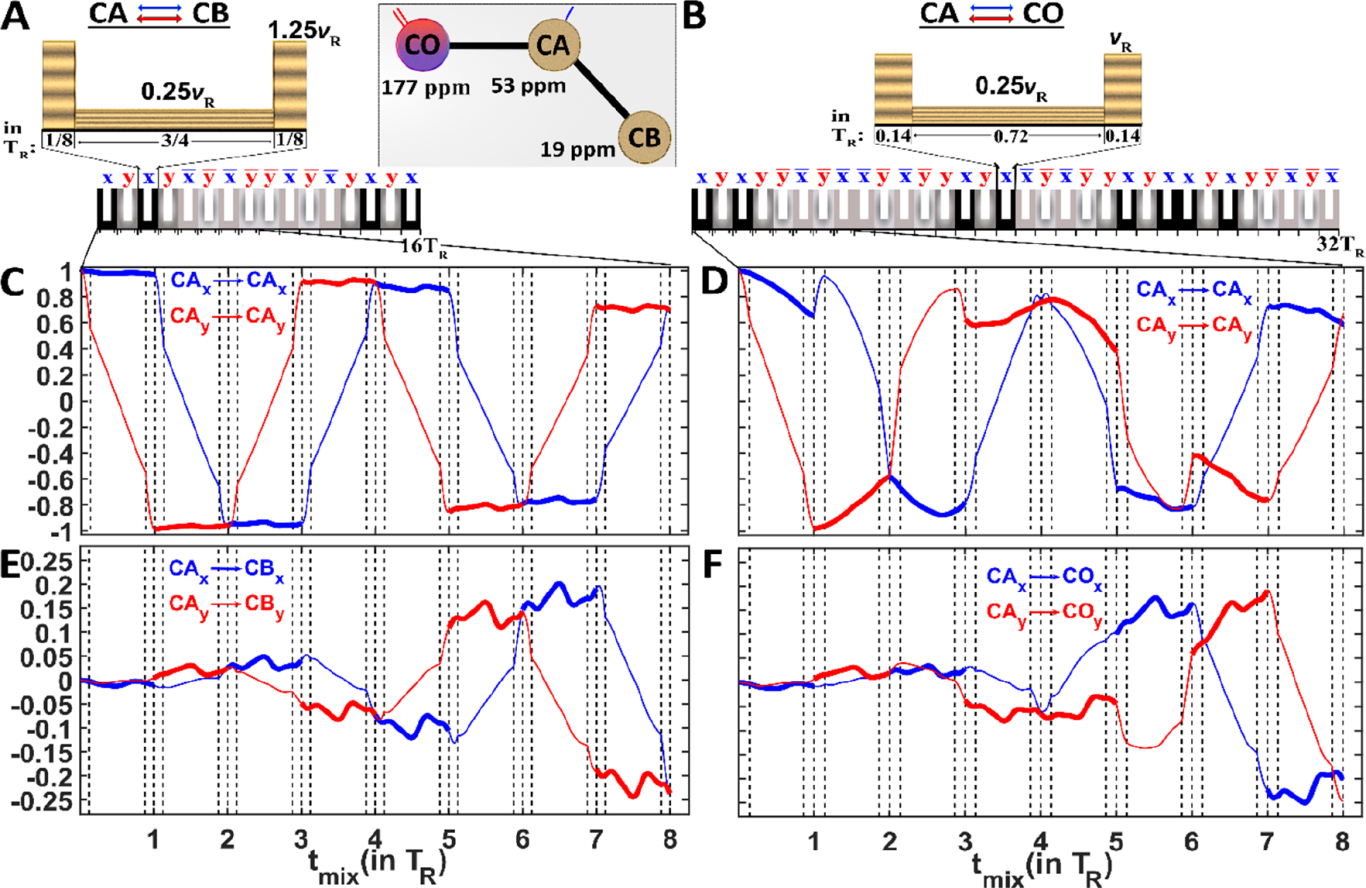
(A) hSPEPS^CA–CB^ and (B) hSPEPS^CA–CO^ sequences for carbon aliphatic–aliphatic and CA–CO transfers. Each repeated hSPEPS element consists of (A) 16 amplitude-modulated pulses and (B) 32 amplitude-modulated pulses, with each amplitude-modulated pulse having the length of one rotor period (*T*_R_). Each amplitude-modulated pulse consists of three constant-amplitude pulses with the same phase. The ratio of the amplitudes are (A) 100:20:100% for hSPEPS^CA–CB^ and (B) 100:25:100% for hSPEPS^CA–CO^ sequences. (A) For the hSPEPS^CA–CB^ sequence, the optimal rf-field strength is 1.25*ν*_R_ (100% of the amplitude power) regardless of the experimental conditions. (B) For the hSPEPS^CA–CO^ sequence, the optimal rf-field strength varies around *ν*_R_, depending upon experimental conditions. (C–F) Simulated evolution of (C and D) CA_*x*_, CA_*y*_, CB_*x*_, and CB_*y*_ and (E and F) CA_*x*_, CA_*y*_, CO_*x*_, and CO_*y*_ operators during the first eight rotor periods. In the simulations, a three-spin system (*I*_3_, inset in panel A) was used at a 600 MHz external magnetic field and 55 kHz MAS. Signals along *x* and *y* are colored blue and red, respectively. Note that to better illustrate the transfer, the dipolar coupling strength was increased in these simulations. Further details of simulations are presented in the Experimental Methods of the Supporting Information.

To obtain these sequences, we simulated a three-spin system (depicted in the inset of Figure 1A). For carbon CA–CB transfer, we kept the carrier frequency (CF) inside the aliphatic region (at 42 ppm), while for CA–CO transfer, it was set to 113 ppm. Starting from the SPEPS sequence,^41^ we modified the phase and amplitude of the pulses, to preserve maximal transfer efficiency between homonuclear spins under different experimental conditions.

The simulated dependence of sequences upon the experimental conditions is evaluated in Figures S1 and S3–S6 of the Supporting Information. In simulations depicted in Figure S1 of the Supporting Information, hSPEPS^CA–CB^ and hSPEPS^CA–CO^ sequences provide transfer efficiencies of approximately 40 and 43% for each transverse component, respectively. For experiments depicted in Figure S2 of the Supporting Information, hSPEPS^CA–CO^ provides an estimated transfer efficiency of 34% [this is in line with the average estimate when comparing the CANH and CONH signals to the CO(CA)NH and CACONH signals in one dimension (1D)]. This compares favorably to the 42% transfer estimated for a microcrystalline sample using DREAM.^26^

The elements remain efficient under different MAS rates (20–115 kHz) and external magnetic fields (600–1200 MHz), according to the simulations of Figures S3 and S4 of the Supporting Information. For both sequences, the optimal rf-field strength conditions are broad, suggesting a small dependence upon rf-field power missets. For hSPEPS^CA–CB^, the optimal rf-field strength of the higher amplitude is 1.25*ν*_R_, regardless of the experimental conditions (left column in Figures S3 and S4 of the Supporting Information). In contrast, for hSPEPS^CA–CO^, the optimal rf-field strength of the higher amplitude varies with changes in either the MAS rate or external magnetic field but is always near *ν*_R_ (right column in Figures S3 and S4 of the Supporting Information). The ratio between high and low amplitude was kept fixed at 5 or 4 for these hSPEPS^CA–CB^ and hSPEPS^CA–CO^ simulations, respectively. These simulations indicate that, for the hSPEPS^CA–CB^ sequence, the required rf-field power can be calculated from a single-pulse calibration, and for the CA–CO sequence, a 1D optimization procedure is sufficient. The dependence of the CA–CB transferred signal on the offset values at a 1200 MHz external magnetic field is evaluated in Figure S5 of the Supporting Information. The potential influence of undesired cross transfers^40^ is addressed in Figure S6 of the Supporting Information.

Panels C–F of Figure 1 reveal the evolution of the transverse operators during the first eight rotor periods of hSPEPS^CA–CB^ (C and E) and hSPEPS^CA–CO^ (D and F), respectively. Some similarity in the evolution of the operators during hSPEPS and SPEPS^41^ sequences is observed. When the initial operator (CA_*x*_, CA_*y*_) has the same phase as the applied rf field and the offset is small (panels C and D of Figure 1), the operator becomes locked (indicated by thick lines). In the case of the CA–CO sequence (panels E and F of Figure 1), the large offset influences the evolution of the initial operators. However, by the end of the eighth rotor period, this influence is almost eliminated and the initial operators, shown in Figure 1D, have almost the same amplitude as the same operators in Figure 1C. For the initial operator with a 90° phase difference compared to that of the applied pulse, the inversion is observed at the end of the rotor period for both sequences (thin lines). However, unlike SPEPS, transfer occurs not only mainly during spin locking; it occurs at the same time for both transverse operators, CA_*x*_ and CA_*y*_ (panels E and F of Figure 1).

To characterize the aliphatic–aliphatic transfer, two-dimensional (2D) (H)CC spectra using hSPEPS^CA–CB^ were recorded at different mixing times. Figure S7 of the Supporting Information displays the resulting spectra of influenza A M2 membrane protein,^47−50^ which were recorded on a 600 MHz spectrometer with 55 kHz MAS. The maximal transfer efficiency of the negative cross peaks is observed with a 1.16 ms mixing time (Figure S7D of the Supporting Information). With mixing times of 1.16 and 1.45 ms (panels D and E of Figure S5 of the Supporting Information), relayed transfer is also observed as positive peaks.^51^Figure S8 of the Supporting Information compares hSPEPS^CA–CB^ to DREAM,^52^ where the former provides higher CB–CA transfer efficiency.

Figures S9 and S10 of the Supporting Information compare hSPEPS to hTROP. Figure S9 of the Supporting Information displays 1D (HCACON)H and (HCOCAN)H spectra comparing hSPEPS^CA–CO^ and hTROP^40^ sequences for the CA–CO and CO–CA transfers. A single rf-field parameter was optimized in each case. The result was higher transfer efficiency with hSPEPS^CA–CO^ for CA–CO transfers compared to hTROP. For CO–CA transfers, both sequences provide similar transfer efficiency. Figure S10 of the Supporting Information shows 2D (H)CC spectra with hSPEPS^CA–CO^ (black) or hTROP (red) to emphasize that hSPEPS simultaneously drives CA → CO and CO → CA transfers, while the transfer is unidirectional for hTROP.

The hSPEPS sequence performs well compared to hTROP and *J*-based transfer. Figure 2 makes three comparisons of ^13^C–^15^N projections from three-dimensional (3D) assignment spectra,^27^ used to link backbone residues with detection at the amide proton. Figure 2A compares hSPEPS and hTROP transfer for the (H)CA(CO)NH experiment. While both methods, hSPEPS and hTROP, generally offer similar transfer efficiency for most peaks, hSPEPS^CA–CO^ appears to be more robust for residues in an environment with higher proton density, as seen in G34, for example (strip 1 in Figure 2A). In Figure 2B, the related 3D (H)CO(CA)NH experiment is used to compare the same hSPEPS sequence to the corresponding hTROP transfer. Note that different hTROP-shaped pulse profiles were used for CA → CO and CO → CA transfers. The shapes were developed by Blahut et al.^40^ for 55 kHz MAS and particular spectrometer frequencies and were downloaded from https://optimal-nmr.net/sequences.html. Again, most peaks demonstrate similar intensities, but several are more intense with hSPEPS, as shown in the selections. Three such strips, 2–4, are shown. In Figure 2C, 3D (H)CB(CA)NH with hSPEPS transfer is compared to the analogous (HCA)CB(CA)NH with an out-and-back *J*-based transfer.^53^Figure 2C demonstrates a clear advantage of hSPEPS^CA–CB^ over the *J*-based sequence when performing CB(CA) transfer for samples with short *T*_2_ relaxation rates. In two selected strips (5 and 6), CB cross peaks are evident in the hSPEPS^CA–CB^ spectrum but are still obscured by the noise in the *J*-based spectrum.

**Figure 2:**
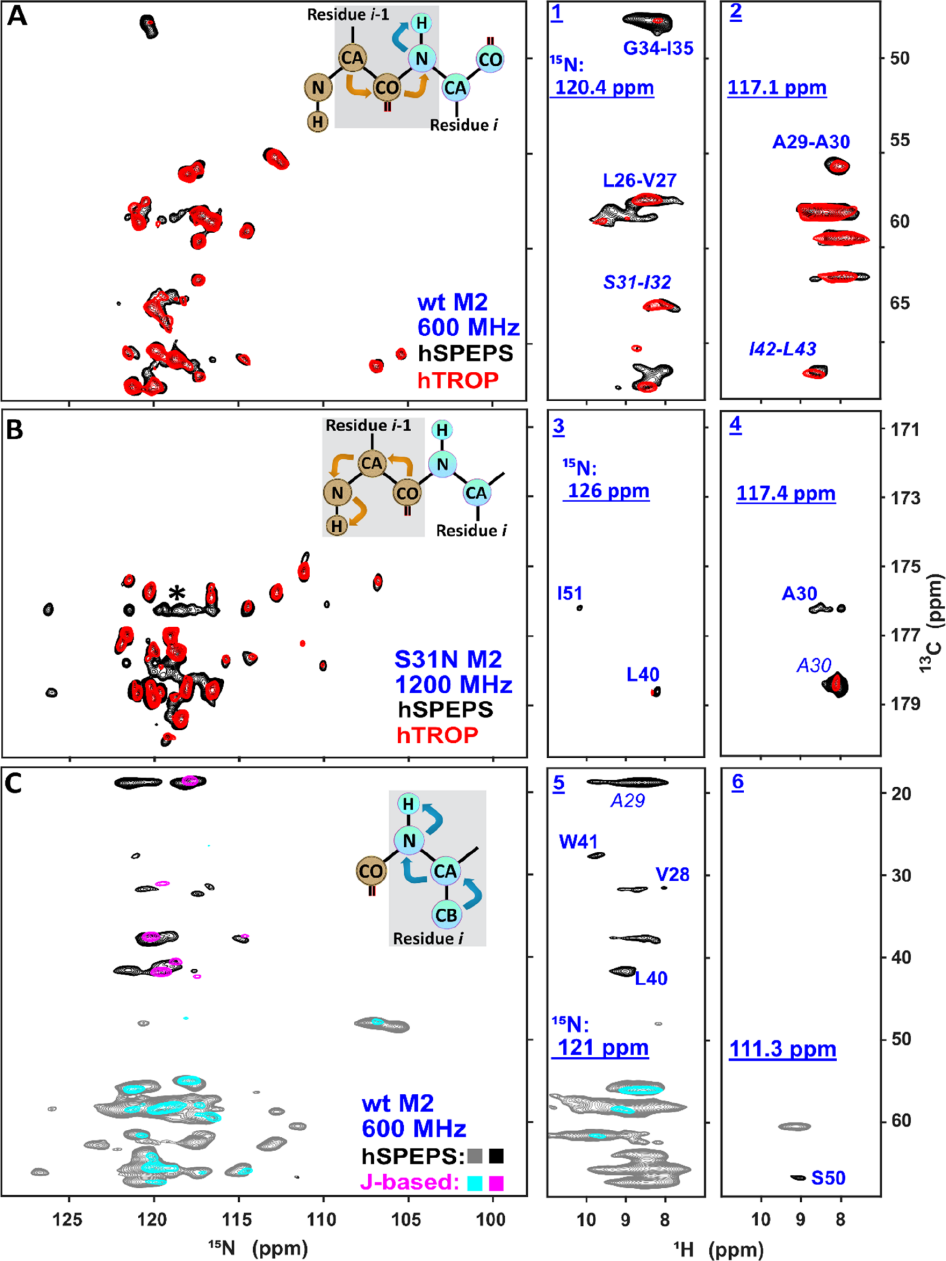
Performance of hSPEPS compared to hTROP and *J*-based transfer. In each case, the ^15^N–^13^C correlation spectrum shows a projection from a 3D spectrum, and the ^13^C–^1^H correlations are selected strips from 3D, at the indicated ^15^N frequency. (A) Comparison of hSPEPS^CA–CO^ (black, 1.16 ms mixing) and hTROP^CA→CO^ (red, 1.8 ms mixing) for CA → CO transfer in 3D (H)CA(CO)NH spectra. During hSPEPS^CA–CO^ and hTROP^CA→CO^ sequences, the carrier frequency was set to 113.7 and 53.7 ppm, respectively. The data were recorded with the wild-type (WT) M2 sample, at a 600 MHz instrument and 55 kHz MAS. (B) Comparison of hSPEPS^CA–CO^ (black, 1.16 ms mixing) and hTROP^CO→CA^ (red, 1.8 ms mixing) for the CO → CA transfer in (H)CO(CA)NH spectra. During hSPEPS^CA–CO^ and hTROP^CO→CA^ sequences, the carrier frequency was set on 113.7 and 168 ppm, respectively. The data were recorded with the S31N M2 sample at 1200 MHz with 55 kHz MAS. (∗) CF artifacts as a result of echo/anti-echo acquisition. Final pulse programs (see the Supporting Information) use echo/anti-echo-TPPI^54,55^ to move this artifact to the spectrum edges. (C) Comparison of (H)CB(CA)NH using hSPEPS to (HCA)CB(CA)NH using *J*-based CB → CA transfer. Gray (positive) and black (negative) represent hSPEPS^CA–CB^ (1.16 ms mixing), and cyan (positive) and magenta (negative) represent *J*-based transfer (13.8 ms total transfer time). During both the hSPEPS^CA–CB^ and *J*-based sequences, the carrier frequency was set to 39.7 ppm. The data were recorded with the WT M2 sample at a 600 MHz external magnetic field and 55 kHz MAS. Further experimental details are given in the Supporting Information.

A quantitative comparison of the peak intensities of the 3D spectra of Figure 2 is displayed in Figure 3. The red lines are linear fits with the *y* intercept fixed to 0, showing the average improvement in peak intensity. The blue lines show linear fits without fixing the *y* intercept. A positive *y* intercept indicates a particular improvement for weaker peaks, which was also noted for SPEPS.^41^ All errors are reported at 1 standard deviation. In panels A and B, hSPEPS outperforms hTROP by factors of, on average, 1.12 and 1.64, respectively. In panel C, the improvement in the signal-to-noise ratio of hSPEPS over *J*-based hSPEPS reaches a factor of 2.85. Alanine peaks are particularly strong in hSPEPS (Figure 3C). This can be rationalized because there is no loss of signal to CG for alanine. Note that, for the out-and-back *J* transfers, there is theoretically no loss from the CB–CG transfer. It is worth mentioning that we sometimes observed similar intensity with hSPEPS and hTROP. Figure S11 of the Supporting Information depicts two such spectra, yet even in this case, the benefit of hSPEPS was evident for a few peaks.

**Figure 3:**
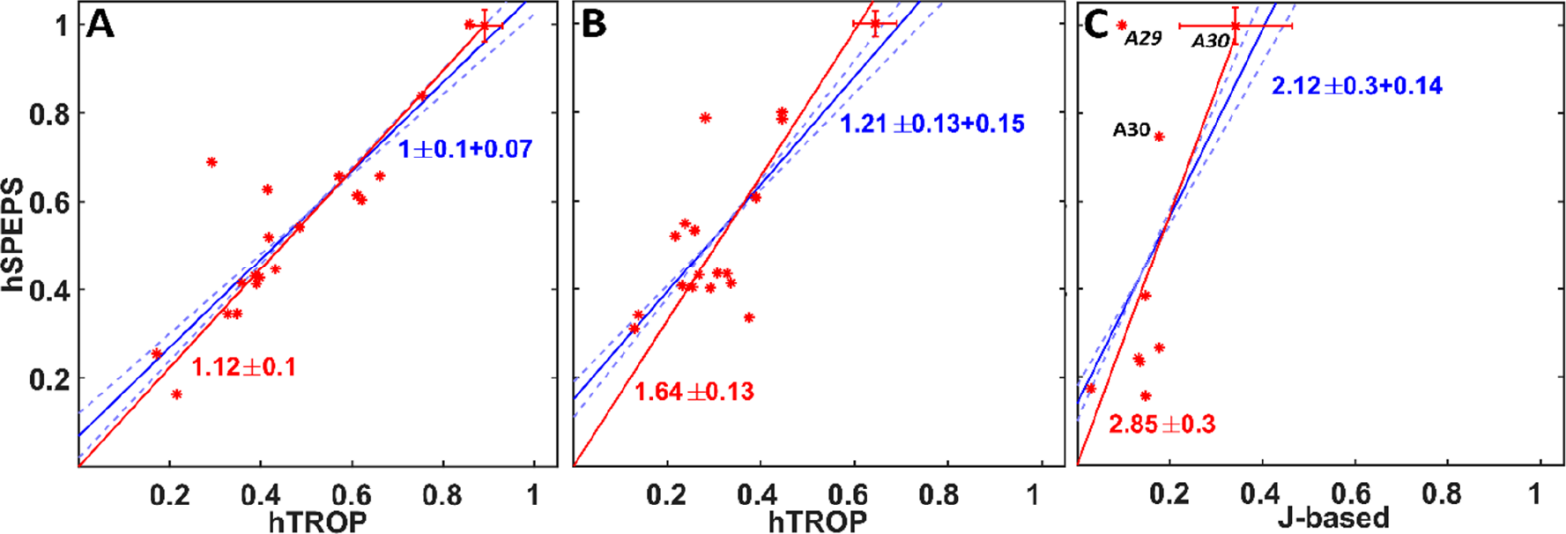
Comparison of the peak intensities from 3D spectra of Figure 2 that utilize (A) CA(CO), (B) CO(CA), or (C) CB(CA) transfer for selected residues: hSPEPS (*y* axis) and hTROP (*x* axis) in panels A and B and hSPEPS (*y* axis) and *J*-based transfer (*x* axis) in panel C. The lines were obtained with linear least squares fitting, either with (red) or without (blue) fixing the intercept to zero. In panels A and C, only assigned and unambiguous peaks were selected. In panel B, all peaks with a signal-to-noise ratio above 10 were selected. The dashed lines were fit with fixed slope values: solid blue lines ± slope error.

We also evaluated the hSPEPS^CA–CO^ sequence for CA–CO transfers in the bacterial rhomboid protease GlpG^56^ using 100 kHz MAS at a 1200 MHz spectrometer (Figure 4). GlpG is a bacterial member of the rhomboid protease family, with intramembrane proteases characterized by a Ser-His catalytic dyad. Solid-state NMR spectra of GlpG in liposomes have been shown to be well-resolved,^56^ which facilitates the detailed investigation of the structure, dynamics,^57,58^ and inhibition^59^ of GlpG.

**Figure 4:**
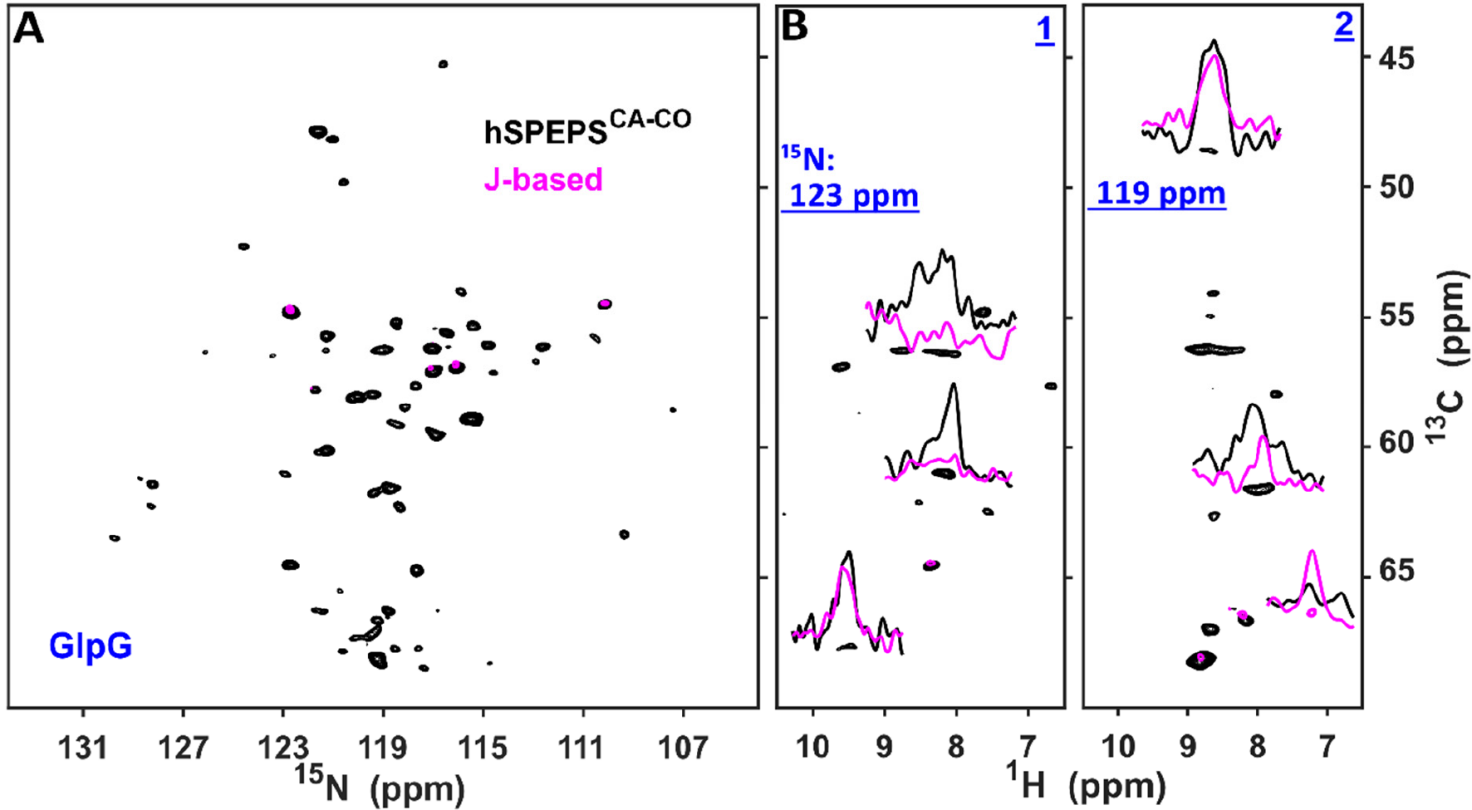
Bacterial rhomboid protease GlpG (H)CA(CO)NH hSPEPS^CA–CO^ was measured with a 1200 MHz spectrometer and 100 kHz MAS. (A) ^13^C–^15^N projections and (B) two strips (1 and 2 at the amide nitrogen frequencies) extracted from 3D spectra: (H)CA(CO)NH with hSPEPS^CA–CO^ (black, 0.96 ms transfer time) and (HCO)CA(CO)NH using *J*-based transfer (magenta, 2 times 6.67 ms transfer time). The (HCO)CA(CO)NH spectrum was recorded with 72 scans, while (H)CA(CO)NH was collected with 24 scans. In all figures, the magenta spectrum is normalized by a factor of 3 (the ratio of scan). Further experimental details are given in the Supporting Information.

Figure 4A compares the ^13^C–^15^N projection of a 3D (H)CA(CO)NH spectrum recorded with hSPEPS (black, 0.96 ms mixing) to the 3D (HCO)CA(CO)NH spectrum recorded using *J*-based carbon transfer^27^ (magenta). For the *J* transfer, the optimal transfer time was calculated on the basis of the bulk *T*_2′_ relaxation time of CO spins, which was 13 ms. Despite recording 3-fold more data for the *J*-based spectrum, more peaks were identified in the SPEPS-based spectrum. Example comparisons are shown in the two selected strips (strips 1 and 2 in Figure 4B).

In summary, we introduced two homonuclear simplified preservation of equivalent pathway spectroscopy sequences, hSPEPS^CA–CO^ and hSPEPS^CA–CB^, which simultaneously transfer both transverse components. The sequences are straightforward to implement at different spinning frequencies and magnetic fields and can be quickly optimized by varying a single rf-field parameter and then the mixing time. The sequences are tolerant to rf-field missets. We tested the performance of SPEPS with membrane protein samples, incorporating the elements into 3D (H)CA(CO)NH, (H)CO(CA)NH, and (H)CB(CA)NH protein assignment experiments. hSPEPS elements demonstrated high performance for excitation of backbone and side-chain carbon–carbon correlations. We anticipate that these elements will be routinely used in protein amino acid assignment experiments for challenging biological samples at fast and ultrafast MAS rates.

## Supporting Information Available

Numerical simulations of hSPEPS, additional experimental data using hSPEPS, DREAM, and hTROP for dipolar recoupling, experimental parameters, and Bruker TopSpin pulse programs implementing the hSPEPS sequence (PDF)
hSPEPS^CA–CO^ shape (TXT)
hSPEPS^CA-CB^ shape (TXT)
