# Supplementary material for: Homonuclear Simplified Preservation of Equivalent Pathways Spectroscopy
Source: J Phys Chem Lett. 2024 Jun 10;15(24):6272–8. doi: 10.1021/acs.jpclett.4c00991 (PMC11194807; doi:10.1021/acs.jpclett.4c00991)
Supplement: Supplementary file 1 — jz4c00991_si_001.pdf [file jz4c00991_si_001.pdf]

Homonuclear Simplified Preservation of Equivalent Pathways Spectroscopy

Authors: Evgeny Nimerovsky[1], Spyridon Kosteletos[2], Sascha Lange[2], Stefan Becker[1], Adam Lange[2] & Loren B. Andreas[1]**

[1]Department of NMR based Structural Biology, Max Planck Institute for Multidisciplinary Sciences, Am Fassberg 11, Göttingen 37077, Germany

[2] Department of Molecular Biophysics, Leibniz-Forschungsinstitut für Molekulare Pharmakologie, Robert-Rössle-Straße 10, Berlin 13125, Germany

*Corresponding authors: land@mpinat.mpg.de ORCID: 0000-0003-3216-9065 and evni@mpinat.mpg.de ORCID: 0000-0003-3002-0718.

Contents

SIMULATIONS AND EXPERIMENTS S2
EXPERIMENTAL METHODS S21
  Simulations S21
  Sample Preparation S22
    Influenza A M2 samples S22
    Bacterial rhomboid protease GlpG S22
Solid State NMR Spectroscopy S23
  hSPEPS sequence set up S23
  Artifacts in multi-dimensional experiments S23
  Sequences and experimental parameters S24
  Compiling script (Echo/ Anti-Echo mode into STATES/STATES-TPPI mode). S40
BRUKER PULSE PROGRAMS S40
  2D (H)CC S40
  2D (H)CACO S43

3D (H)CA(CO)NH........ S45
3D (H)CO(CA)NH........ S51
3D m(H)CO(CA)NH........ S56
3D (H)CB(CA)NH........ S62
REFERENCES........ S67

SIMULATIONS AND EXPERIMENTS

The hSPEPS$^{CA\text{-}CB}$ sequence for carbon aliphatic-aliphatic transfers (depicted in Figure 1A in the main text, labeled as CA-CB) consists of sixteen amplitude-modulated pulses[1,2] with a total length of 16 rotor periods ($16T_R$) and the following XY phase-cycling:[3] $xyxy\bar{x}\bar{y}\bar{x}\bar{y}\bar{y}\bar{x}\bar{y}\bar{x}yxyx$. Each amplitude-modulated pulse is comprised of the following amplitude values of $[1.25\nu_R; 0.25\nu_R; 1.25\nu_R]$ (where ν_R is the MAS rate: $\nu_R = 1/T_R$) with lengths of $\left[\frac{1}{8}T_R; \frac{6}{8}T_R; \frac{1}{8}T_R\right]$, respectively. The total flip angle of each amplitude-modulated pulse is 180°. The sequence is repeated as necessary to reach the desired mixing time.

The hSPEPS$^{CA\text{-}CO}$ sequence for CA-CO transfers (depicted in Figure 1B, labeled as CA-CO) consists of thirty two amplitude-modulated pulses[1,2] with a total length of 32 rotor periods ($32T_R$) and the following XY phase-cycling:[3] $xyxy\bar{y}\bar{x}\bar{y}\bar{x}\bar{x}\bar{y}\bar{x}\bar{y}yxyx\bar{x}\bar{y}\bar{x}\bar{y}yxyxxyxy\bar{y}\bar{x}\bar{y}\bar{x}$. Each amplitude-modulated pulse comprises three pulses with following **approximate** amplitude values of $[\nu_R; 0.25\nu_R; \nu_R]$ (where ν_R is the MAS rate: $\nu_R = 1/T_R$) and lengths of $[0.14T_R; 0.72T_R; 0.14T_R]$, respectively. The total flip angle of the single amplitude-modulated pulse is approximately 165.5°.

Figure S1 depicts simulated CA-CB (Figure S1A) and CA-CO (Figure S1B) curves, using hSPEPS$^{CA\text{-}CB}$ and hSPEPS$^{CA\text{-}CO}$ sequences (depicted in Figure 1A and B in the main text). The elements are double quantum sequences,[4] such that transferred signals are detected with negative amplitude. The simulated transfer efficiencies reach approximately 40% and 43% for

CA-CB (A, C) and CA-CO (B, D) sequences, respectively. In (A) and (C), $CA_x \rightarrow CB_x$ and $CA_y \rightarrow CB_y$ curves are almost identical. In (B) and (D) there are differences in $CA_x \rightarrow CO_x$ and $CA_y \rightarrow CO_y$ curves. To eliminate this difference, compensatory phase cycling is applied, shifting all hSPEPS pulses by 90° in another scan, similar to the case of heteronuclear SPEPS.[5] The measured signals become the average signals of two transverse paths: $CA \rightarrow CB = (CA_x \rightarrow CB_x + CA_y \rightarrow CB_y)/2$ and $CA \rightarrow CO = (CA_x \rightarrow CO_x + CA_y \rightarrow CO_y)/2$. In the subsequent figures, Figures S3-S4, for simplicity, we plot the average signals.

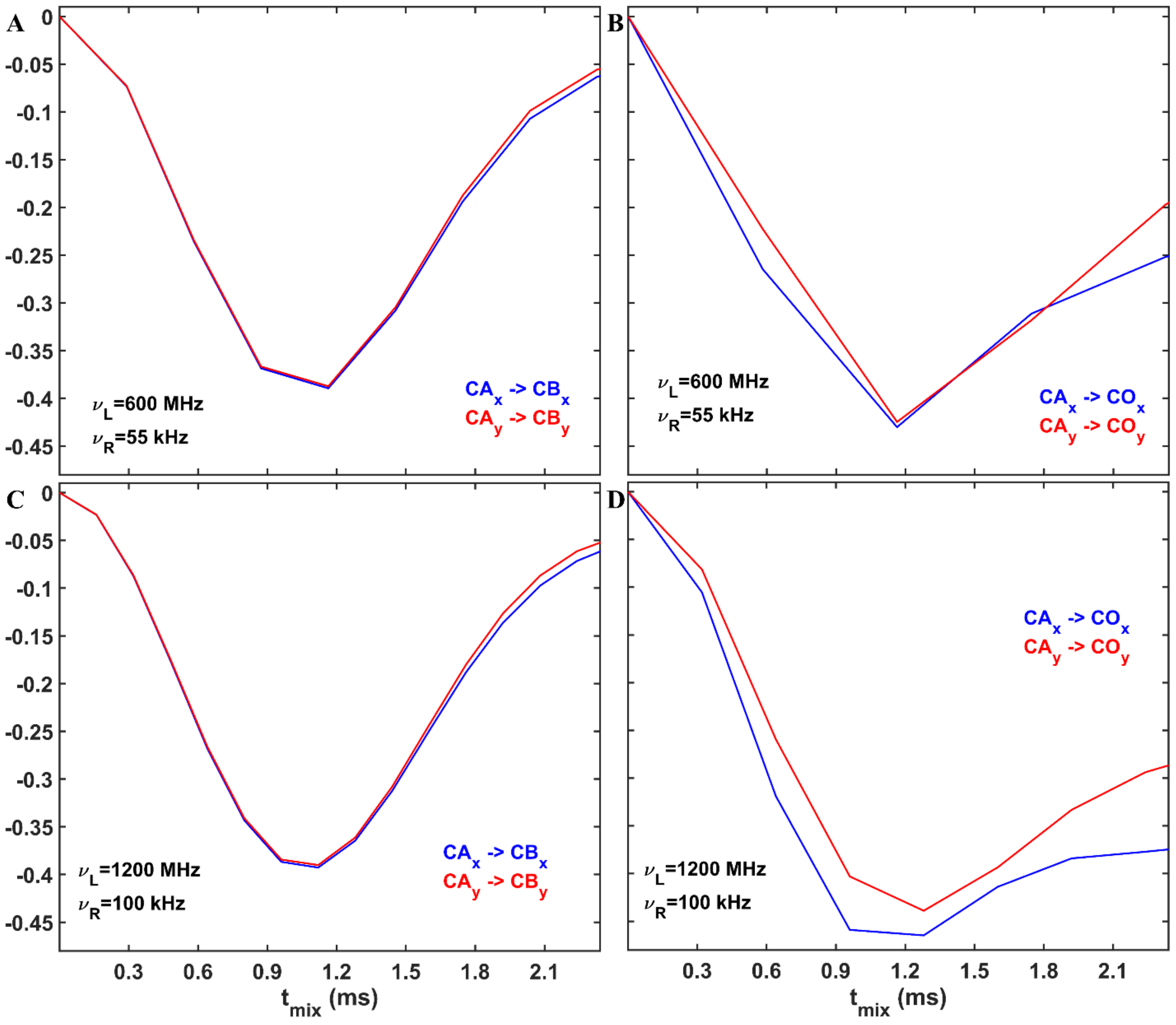

Figure S1 Simulated hSPEPS curves for CA-CB (A, C) and CA-CO (B, D) transfers. The x- and y-components of transferred magnetization are plotted individually, as indicated. In (A-B), the conditions were a 600 MHz spectrometer using 55 kHz MAS. The carrier frequency was set to 42 ppm. In (C-D), simulations correspond to a 1200 MHz spectrometer with 100 kHz MAS. The carrier frequency (CF) was set to 113 ppm. In the simulations, a three-spin system (I_3) was used with homonuclear dipolar coupling values of 2.13 kHz, 0.48 kHz and 2.12 kHz between CO-CA, CO-CB and CA-CB, respectively. The isotropic chemical shifts and chemical shift anisotropy (in ppm) were [177;53;19] and [110;70;60], respectively.

Due to differences in initial carbon polarization, it is not straightforward to determine the experimental CA-CO transfer efficiency. In Figure S2, we show the spectra used to roughly estimates the CA-CO transfer efficiency by calculating the ratio between the carbonyl signal after hSPEPS$^{CA\text{-}CO}$ transfer (the area between 168 ppm and 179.59 ppm in the black spectrum) and the initial aliphatic signal (the area between 46.27 ppm and 68.6 ppm in the gray spectrum). This ratio is 38%, in good agreement with the simulated transfer efficiency. The transferred signal may contain a small contribution from direct CB-CO transfers and therefore, the ratio may slightly overestimate. On the other hand, CB signal from Ser and Thr residues are within the integrated CA signal region, leading to underestimation of the transfer efficiency.

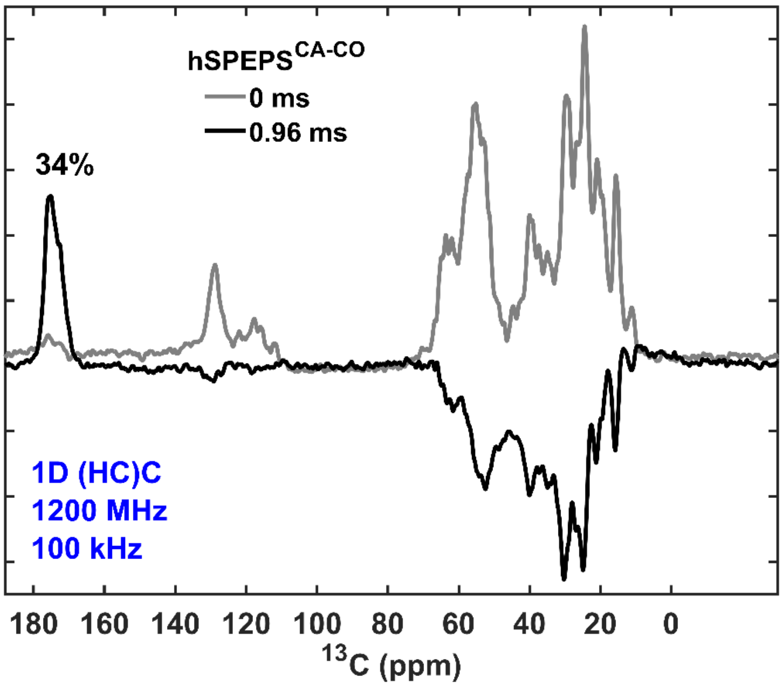

Figure S2 1D (HC)C spectra of Bacterial rhomboid protease GlpG with zero hSPEPS$^{CA\text{-}CO}$ mixing time (gray) and 0.96 ms hSPEPS$^{CA\text{-}CO}$ mixing time (black). The black spectrum was inverted with respect to the gray spectrum. The spectra were acquired at a 1200 MHz spectrometer with 100 kHz MAS. The initial proton-carbon transfer was implemented with 0.7 ms CP with an 80%-100% amplitude ramp[6] applied on the ^{1}H channel (118 to 147 kHz) and 28 kHz on the ^{13}C channel.

Figure S3 depicts the dependence of average transfer efficiency of hSPEPS$^{CA\text{-}CB}$ (left column) and hSPEPS$^{CA\text{-}CO}$ (right column) sequences on rf-field strengths and external magnetic fields under three different MAS rates: 55 kHz – upper; 75 kHz – middle and 100 kHz – lower. For both sequence, the optimal conditions are broad, which suggest a small dependence on rf-field power missets.

For CA-CB transfers (left column), the location of the optimal rf-field strength ($1.25\nu_R$) has a small dependence on the experimental parameters – the MAS rate and external magnetic fields. Regardless of the experimental conditions, the optimal rf-field strength is set to $1.25\nu_R$ (gray dashed line). The required rf-field power can be calculated from a single pulse calibration. Such

behavior is very useful for detecting Double Quantum transfers between spins inside the same band. In this case, a routine 1D optimization procedure for finding the optimal rf-field power is not efficient, since the total measured signal is the sum of the positive initial and negative transferred signals.

For CA-CO transfers (right column), the location of the optimal rf-field strength has a substantial dependence on the experimental parameters. The optimal rf-field strength is near ν_R (gray dashed line), but depends on the MAS rate and external magnetic field. For each set of experimental parameters, the optimal rf-field strength can be easily determined from the maximal detected signal using a routine 1D optimization procedure, for example, in the proton detected sequences (HCACON)H or (HCOCAN)H. For this, the CP conditions should be carefully chosen such that the detected signal consists solely of the transferred signal. In this case, zero (HCACON)H and (HCOCAN)H signals should be observed with zero hSPEPS mixing time. Alternatively, selective pulses can be used to ensure that the starting signal is purely CA or CO.

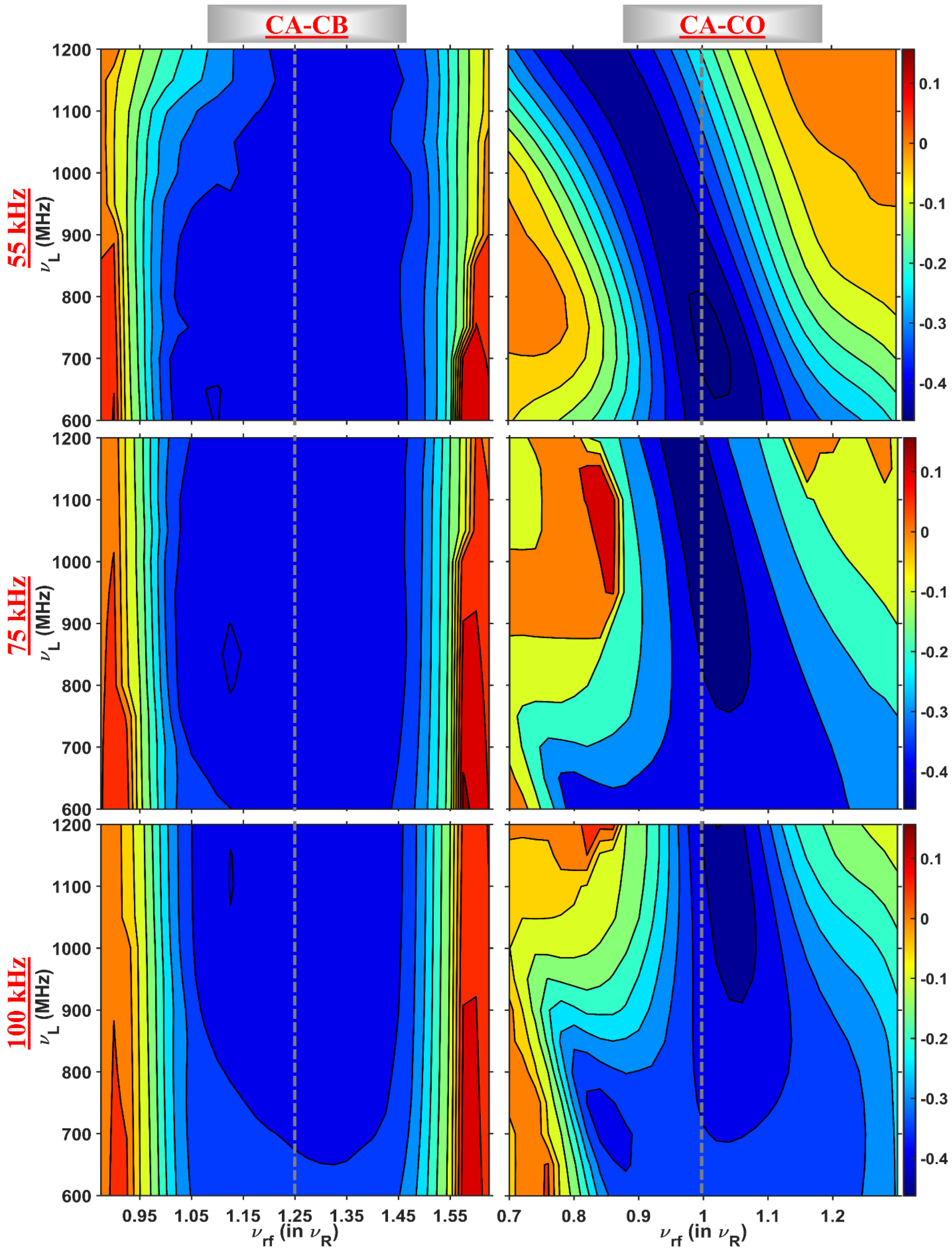
CA-CB
CA-CO
55 kHz
75 kHz
100 kHz
ν_L (MHz)
ν_{rf} (in ν_R)
1200
1100
1000
900
800
700
600
0.95
1.05
1.15
1.25
1.35
1.45
1.55
0.7
0.8
0.9
1
1.1
1.2
0.1
0
-0.1
-0.2
-0.3
-0.4

Figure S3 Simulated average (of both transverse components) CA-CB (left column, 42 ppm CF set) and CA-CO (right column, 113 CF set) transfer efficiency as a function of external magnetic field (ν_L) and rf-field strength (ν_{rf} in units of the MAS rate) for three different MAS rates: 55 kHz – the upper row; 75 kHz – the middle row and 100 kHz – the lower row. In the simulations, a three-spin system (I_3) was used with homonuclear dipolar coupling values of 2.13 kHz, 0.48 kHz and 2.12 kHz between CO-CA, CO-CB and CA-CB, respectively. The isotropic chemical shifts and chemical shift anisotropy (in ppm) were [177;53;19] and [110;70;60], respectively.

Figure S4 depicts the dependence of average transfer efficiency of hSPEPS$^{CA\text{-}CB}$ (A and C) and hSPEPS$^{CA\text{-}CO}$ (C and D) sequences on rf-field strengths and MAS rates under two different external magnetic fields: 600 MHz – A and B; 850 MHz –C and D. At 600 MHz (A), hSPEPS$^{CA\text{-}CB}$ becomes efficient starting with a 20 kHz MAS, while at 850 MHz (B), the minimal MAS rate is 30 kHz. For hSPEPS$^{CA\text{-}CO}$, the minimal MAS rates are 25 kHz (B) and 35 kHz (D).

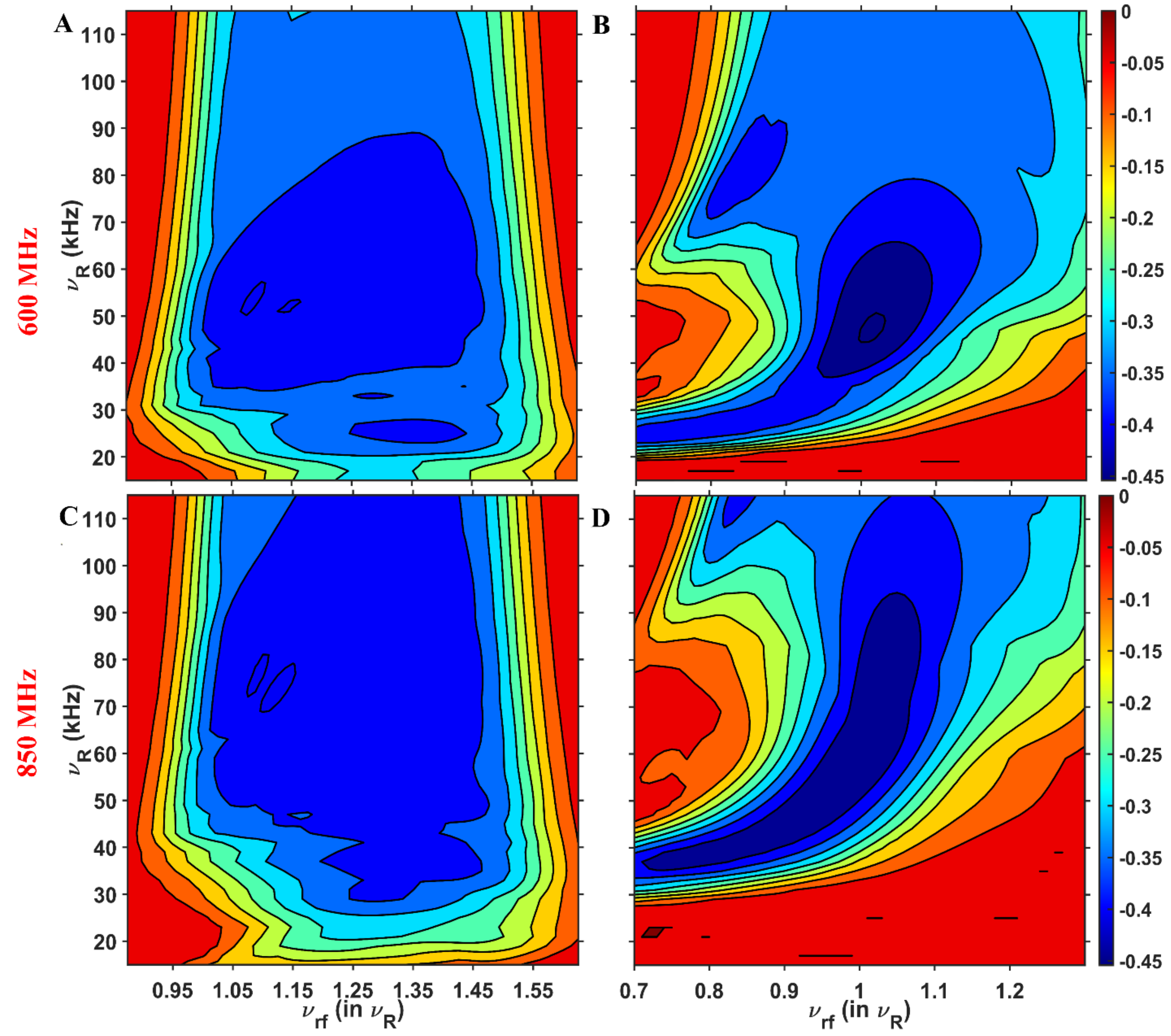

Figure S4 Simulated average (of both transverse components) CA-CB (A and C, 42 ppm CF set) and CA-CO (B and D, 113 CF set) transfer efficiency as a function of MAS rate (ν_R) and rf-field strength (ν_{rf} in units of the MAS rate) for two different external magnetic fields: 600 MHz – A, B and 850 MHz –C, D. In the simulations, a three-spin system (I_3) was used with homonuclear dipolar coupling values of 2.13 kHz, 0.48 kHz and 2.12 kHz between CO-CA, CO-CB and CA-CB, respectively. The isotropic chemical shifts and chemical shift anisotropy (in ppm) were [177;53;19] and [110;70;60], respectively.

The simulated offset dependence of hSPEPS$^{CA\text{-}CB}$ at fixed mixing time is explored in Figure S5. Both the initial spin offset (y-axis) and measured spin offset (x-axis) are varied, with

two different MAS rates. The external magnetic field was set to 28 T (a 1200 MHz spectrometer). While, in general, we observe mixing of signals between spins inside the entire aliphatic region (blue region), the intensities of these signals do decrease towards the extreeme edges of the aliphatic region. For example, at 55 kHz, the transferred signal between initial and final spins inside 60-70 ppm (labeled with a red star in Figure S5A) is about 2.5 times lower compared to the maximum. Therefore, the cross peak between CA-CB spins in Serine amino acid may have weaker peak intensities compared to the cross peaks of other amino acids. This issue is alleviated when spinning at 100 kHz (Figure S5B)

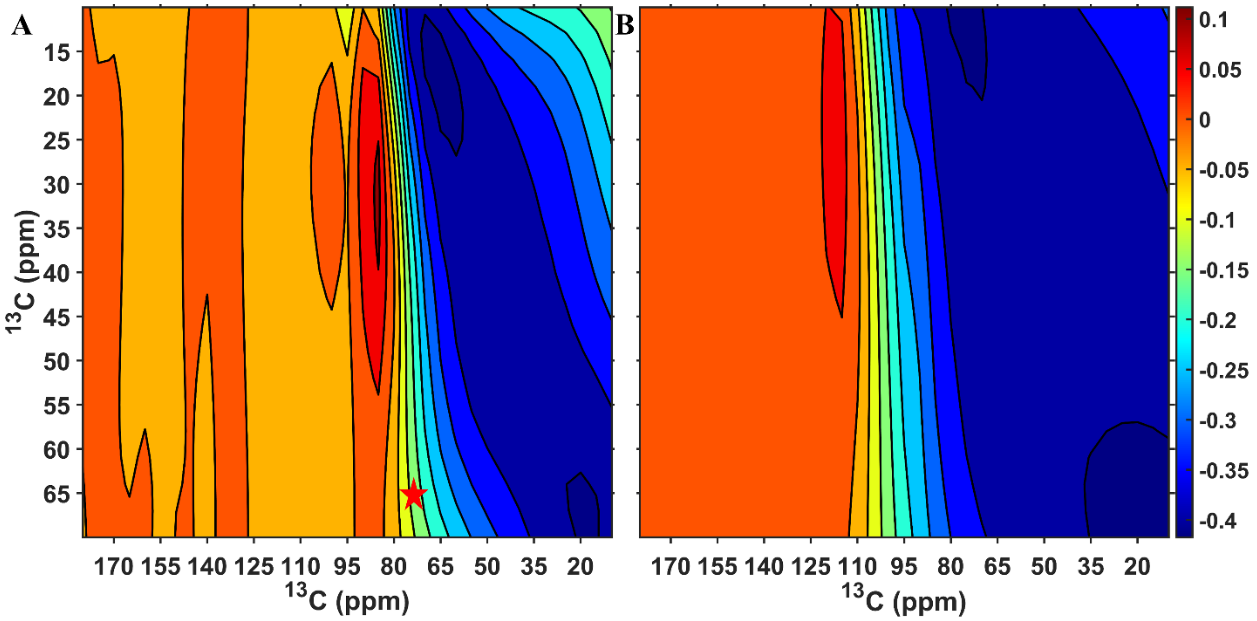

Figure S5 Simulated average (of both transverse components) CA-CB signal at fixed mixing time (1.1636 ms in A and 1.12 ms in B) as a function of carbon offset values at 55 kHz MAS (A) and 100 kHz MAS (B). The carrier frequency was set to 42 ppm and a 28 T external magnetic field was used (1200 MHz spectrometer). In the simulations, a three-spin system (I_3) was used with homonuclear dipolar coupling values of 2.13 kHz, 0.48 kHz and 2.12 kHz between CO-CA, CO-CB and CA-CB, respectively. The isotropic chemical shifts (in ppm) were [177; axis y; axis x]. The chemical shift anisotropy values were [110; 70; X], where X=120 ppm in the carbonyl region; X=190 ppm in the aromatic region and X=65 ppm in the aliphatic region. The star in panel (A) marks a transferred signal for spins with similar isotropic chemical shift values for CA and CB spins as in serine amino acid.

So far we have shown the simulations, where we have considered the average signals of two transverse paths operators: $C_x \rightarrow C'_x$ and $C_y \rightarrow C'_y$. In the following paragraph we consider the influence of the undesired cross-tranfers:[7] $C_x \rightarrow C'_y$ and $C_y \rightarrow C'_x$. In general, the initial density matrix before hSPEPS sequence can be defined as follows:

$$\rho_{before\ hSPEPS} = f_{E/AE} C_x cos(\omega_c t_1) + C_y sin(\omega_c t_1), \qquad \text{Eqn. (S1)}$$

where the appearance two transverse components occurs due to chemical shift evolution of C_x operator, not due to pulse preparation. $f_{E/AE} = \begin{cases} 1, for\ each\ odd\ t_1\ scan \\ -1, for\ each\ even\ t_1\ scan \end{cases}$, which represents evolution of multi-dimensional experiment in Echo / Anti-Echo mode.[8] This difference in sign is achieved with a pair of the 90°-pulses before the hSPEPS sequence, where the phase of the second 90°-pulse is shifted by π every even scan in the indirect dimension (t_1). To consider the influence of these undesired cross-transfers, we have independently consider all four possible transfers, defining four transferred amplitudes: a_{xx}, a_{xy}, a_{yx} and a_{yy}. The amplitudes a_{xx} and a_{yy} are the amplitudes of the desired transfers between operators $C_x \rightarrow C'_x$ and $C_y \rightarrow C'_y$, respectively. The amplitudes a_{xy} and a_{yx} are the amplitudes of the undesired cross-transfers between operators $C_x \rightarrow C'_y$ and $C_y \rightarrow C'_x$, respectively.

The density matrix after hSPEPS sequence can be written as follows:

$$\rho_{after\ hSPEPS} = f_{E/AE} \left[a_{xx} C'_x + a_{xy} C'_y\right] cos(\omega_c t_1) + \left[a_{yy} C'_y + a_{yx} C'_x\right] sin(\omega_c t_1). \qquad \text{Eqn. (S2)}$$

The real and imaginary parts in t_1 dimension is defined as follows:

$$\rho_{real} = \rho_{after\ hSPEPS}(f_{E/AE} = 1) - \rho_{after\ hSPEPS}(f_{E/AE} = -1), \qquad \text{Eqn. (S3A)}$$

$$\rho_{imag} = \rho_{after\ hSPEPS}(f_{E/AE} = 1) + \rho_{after\ hSPEPS}(f_{E/AE} = -1).$$

Eqn. (S3B)

Substituting Eqn. (S2) into Eqns. (S3A-B), the modified Eqns. (S3A-B) are:

$$\rho_{real} = 2\left[a_{xx}C'_x + a_{xy}C'_y\right] cos(\omega_c t_1),$$ Eqn. (S4A)

$$\rho_{imag} = 2\left[a_{yy}C'_y + a_{yx}C'_x\right] sin(\omega_c t_1) = 2\left[a_{yy}C'_y - a_{yx}C'_x\right] sin(\omega_c t_1) + +\left[a_{xy} + a_{yx}\right]C'_x sin(\omega_c t_1).$$ Eqn. (S4B)

Eqns. (S4A-B) can be simplified if we take into account the compensatory phase cycling of hSPEPS sequence. The compensatory phase cycling ($\varphi_{COMP} = 0, 0.5\pi$) eliminates the difference between a_{xx} and a_{yy}, shifting all hSPEPS pulses by 90° in another scan. In this case, the measured signals become the average signals of two transverse paths $a = (a_{xx} + a_{yy})/2$. According to this, Eqns. (R6) can be written as follows:

$$\rho_{real} = 2\left[aC'_x + a'_{xy}C'_y\right] cos(\omega_c t_1),$$ Eqn. (S5A)

$$\rho_{imag} = 2\left[aC'_y - a'_{xy}C'_x\right] sin(\omega_c t_1) + 2\left[a'_{xy} + a'_{yx}\right]C'_x sin(\omega_c t_1),$$ Eqn. (S5B)

where $a'_{xy} = [a_{xy}(\varphi_{COMP} = 0) + a_{xy}(\varphi_{COMP} = 0.5\pi)]/2$ and $a'_{yx} = [a_{yx}(\varphi_{COMP} = 0) + a_{yx}(\varphi_{COMP} = 0.5\pi)]/2$.

Eqns. (S5A-B) can be simplified by rotation with the following unitary operator:

$$U_{\varphi a} = exp\{-i\varphi_a C'_z\},$$ Eqn. (S6)

$$\varphi_a = tan^{-1}\left(a_{xy}/a\right)$$

The modified Eqns. (S5A-B) after rotation are:

$$\rho'_{real} = U_{\varphi a}^{-1}\rho_{real}U_{\varphi a} \approx 2\sqrt{a^2 + a'^2_{xy}}C'_x cos(\omega_c t_1),$$ Eqn. (S7A)

Eqn. (S7B)

$$\rho'_{imag} = 2\sqrt{a^2 + a'^2_{xy}}C'_y sin(\omega_c t_1) + U^{-1}_{\varphi a} 2[a'_{xy} + a'_{yx}]U_{\varphi a}C'_x sin(\omega_c t_1).$$

If $a'_{xy} + a'_{yx} = 0$, the undesired transfers will have only influence on the phase of the measured signal, which can be easily adjusted with 0th order phase correction function.

Under these conditions, Eqn. (S7) can be written as follows:

$$\rho'_{real} \approx 2\sqrt{a^2 + a'^2_{xy}}C'_x cos(\omega_c t_1) = 2a'C'_x cos(\omega_c t_1), \quad \text{Eqn. (S8A)}$$

$$\rho'_{imag} \approx 2\sqrt{a^2 + a'^2_{xy}}C'_y sin(\omega_c t_1) = 2a'C'_y sin(\omega_c t_1). \quad \text{Eqn. (S8B)}$$

The following Figure S6 shows the simulated $(a'_{xy} + a'_{yx})$ transfers as a function of the external magnetic field and rf-field strength at a 55 kHz MAS for CA-CB (Figure S6A) and CA-CO (Figure S6B) transfers. As can be seen, for hSPEPS sequences with compensatory phase cycling, this sum has negligible small values.

While, the undesired cross-transfers have the influence on the amplitudes of the measured signals (Eqns. S8A-B), they do not generate any peak distortions.

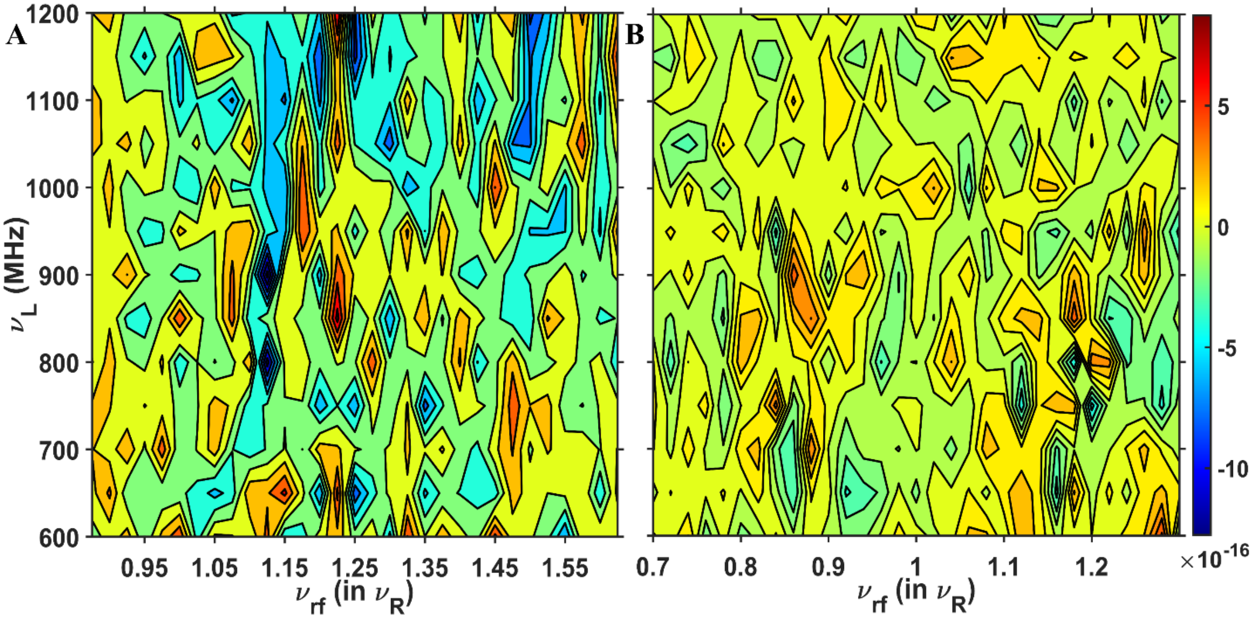

Figure S6 Simulated sum of the undesired $(a_{xy} + a_{yx})$ transfers for CA-CB (A, 42 ppm CF set) and CA-CO (B, 113 CF set) transfers as a function of external magnetic field (ν_L) and rf-field strength (ν_{rf} in units of the MAS rate) at a 55 kHz MAS. In the simulations, a three-spin system (I_3) was used with homonuclear dipolar coupling values of 2.13 kHz, 0.48 kHz and 2.12 kHz between CO-CA, CO-CB and CA-CB, respectively. The isotropic chemical shifts and chemical shift anisotropy (in ppm) were [177;53;19] and [110;70;60], respectively.

Figure S7 depicts 2D (H)CC spectra with different hSPEPS$^{CA\text{-}CB}$ mixing times (A-E). While the diagonal is positive (cyan), the cross peaks appear with a negative sign (black). With a short mixing time (Figure S7A, 0.29 ms), only a few cross peaks are detected. The maximal transfer efficiency of hSPEPS$^{CA\text{-}CB}$ is achieved with a 1.16 ms mixing time (Figure S7D), as observed in simulations (Figure S1). With mixing times of 1.16 ms (Figure S7D) and 1.45 ms (Figure S7E), positive cross peaks appear, which are the result of the relayed stransfer.[9] The buildup of the selected slices as a function of mixing time is depicted in Figure S7F.

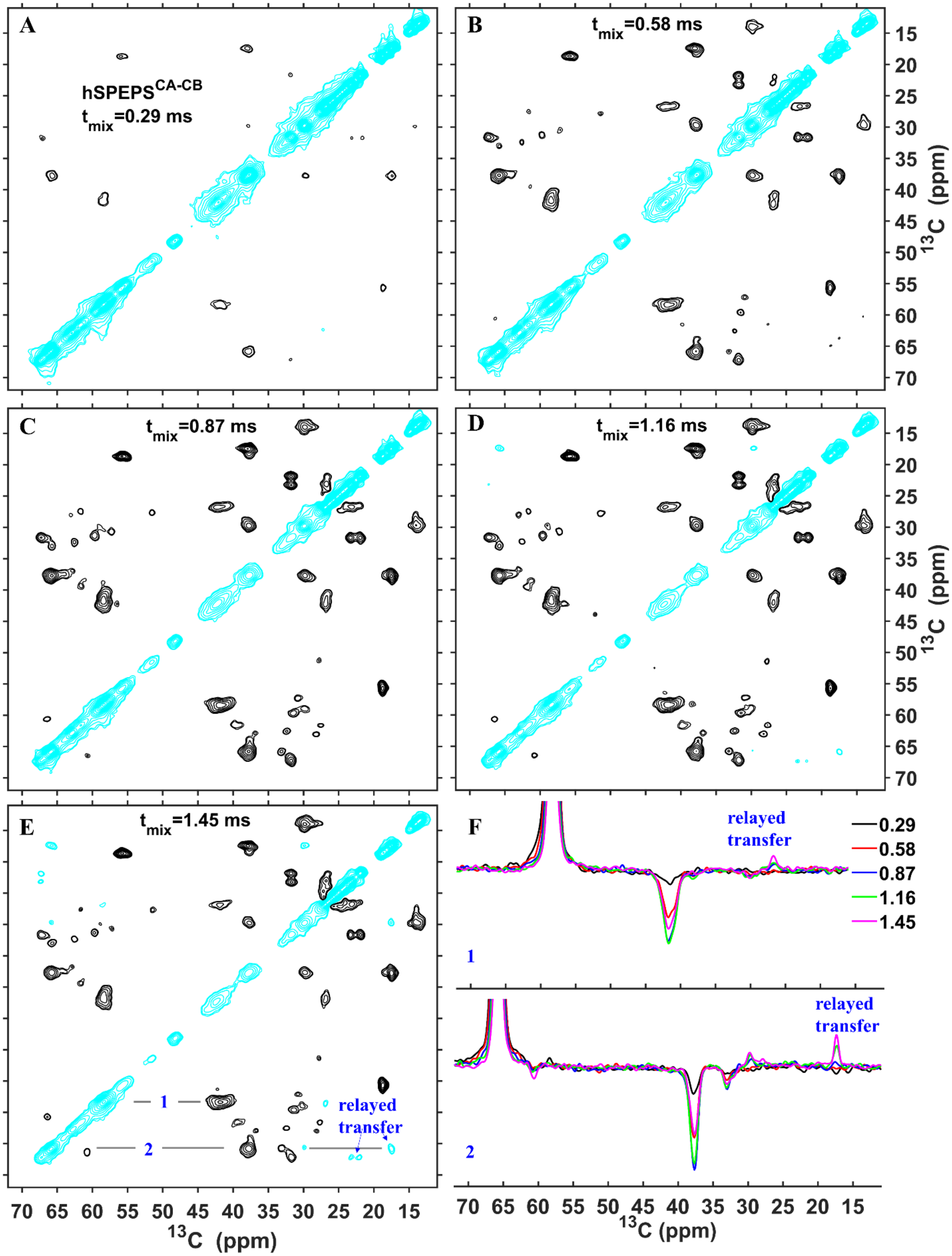

A
hSPEPS^{CA-CB}
t_{mix}=0.29 ms
B
t_{mix}=0.58 ms
C
t_{mix}=0.87 ms
D
t_{mix}=1.16 ms
E
t_{mix}=1.45 ms
1
2
relayed transfer
F
relayed transfer
0.29
0.58
0.87
1.16
1.45
^{13}C (ppm)

Figure S7 2D (H)CC spectra with different hSPEPS$^{CA\text{-}CB}$ mixing times. The spectra were acquired at a 600 MHz spectrometer with 55 kHz MAS using ^{13}C, ^{15}N-labelled WT M2, containing Cu^{2+} ethylenediaminetetraacetic acid to accelerate the acquisition. The positive and negative intensities are depicted with cyan and black contours, respectively. The mixing times are 0.29 ms (A), 0.58 ms (B), 0.87 ms (C), 1.16 ms (D), and 1.45 ms (E). In (F), two slices, recorded at five different mixing times (labeled with 1-2). For hSPEPS$^{CA\text{-}CB}$, 1.25ν_R rf-field strength was applied, which was found, using the calibrated parameters for ^{13}C 90°-pulse: $rf - field\ power_x(in\ Watts) = rf - field\ power_{known}(in\ Watts)\left(\frac{\nu_{rf,x}(in\ kHz)}{\nu_{rf,known}(in\ kHz)}\right)^2$. In all experiments, the carrier frequency was set at 42 ppm. Further details of the experiments are presented below.

Figure S8A depicts 2D (H)CC spectra with DREAM[9–11] (magenta – positive, red-negative) and hSPEPS$^{CA\text{-}CB}$ (cyan – positive, black – negative) sequences. For DREAM, four different spectra with different experimental parameters were collected:

	1st	2nd	3rd	4th
d^{eff} (kHz)	1	1	2	1
Δ (kHz)	11	11	2.75	2.75
t_{mix} (ms)	5	3	3	3

d^{eff} – the effective dipolar coupling; Δ – modulation depth.[10,12]

DREAM with the last experimental parameters (4th) had the highest transfer efficiency among all four, which is shown in Figure S8A. In general, hSPEPS shows better performance for most of the peaks, especially in CA-CB regions. Three selected slices from these spectra are shown in Figure S8B.

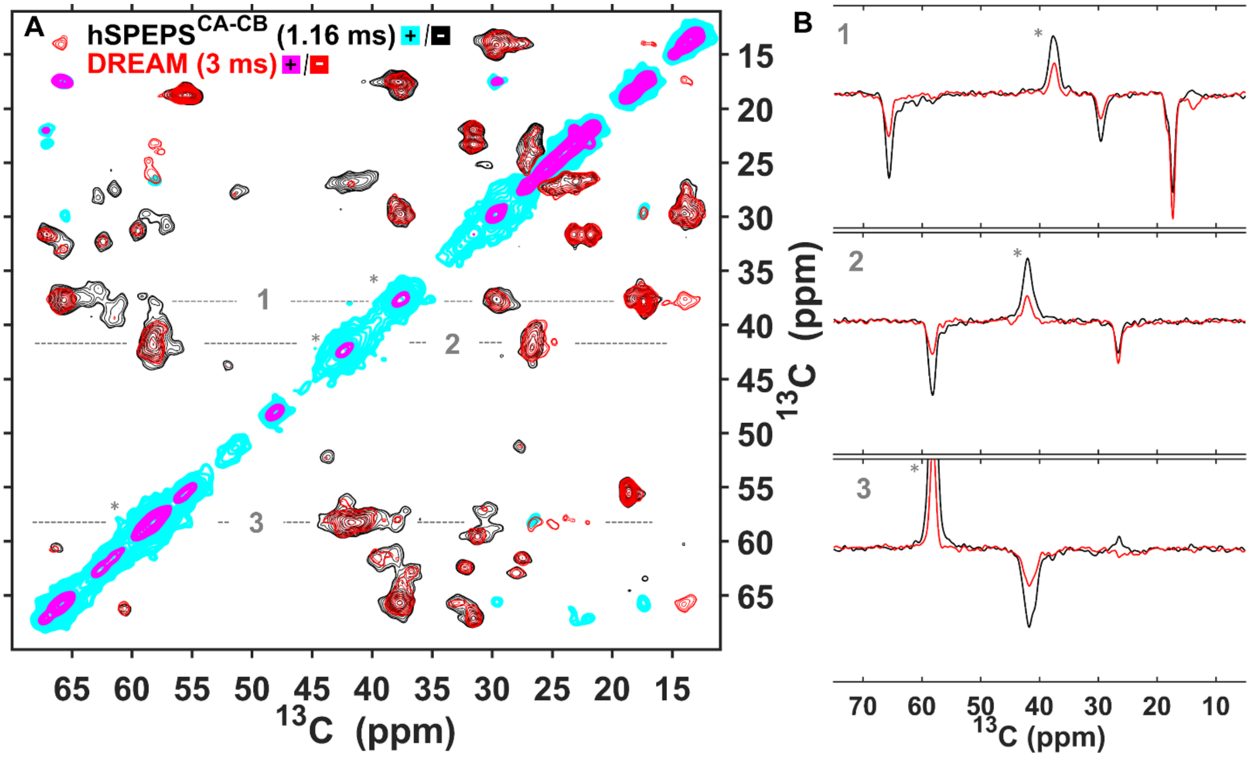

Figure S8 2D (H)CC spectra (A) with hSPEPS$^{CA\text{-}CB}$ (cyan-positive and black-negative) and DREAM (magenta-positive and red-negative) sequences. Three selected slices are shown in B (labeled with 1-3). The gray stars indicate the diagonal peaks. For hSPEPS, 1.16 ms mixing time was used. For DREAM, TAN shape[12] was used with the following input parameters: $d^{eff} = 1$ kHz; $\Delta = 2.75$ kHz and $\bar{\nu}_1 = 27.5$ kHz. In both experiments, carbon carrier frequency was set at 42 ppm. The spectra were acquired at a 600 MHz spectrometer with 55 kHz MAS using ^{13}C, ^{15}N-labelled WT M2, containing Cu^{2+} ethylenediaminetetraacetic acid to accelerate the acquisition.

Figure S9 displays a series of 1D proton-detected spectra, which were acquire to optimize the CA-CO and CO-CA transfers for hSPEPS$^{CA\text{-}CO}$ and hTROP[7] sequences. For hTROP, different shaped pulse profiles were used for CA→CO and CO→CA transfers. The shaped pulse profiles were downloaded from: https://optimal-nmr.net/sequences.html.

Figure S9A,C displays the dependences of hSPEPS$^{CA\text{-}CO}$ (A) and hTROP (C) sequences on rf-field strength at a 600 MHz spectrometer and 55 kHz MAS. Both sequences demonstrate a small dependence on rf-field misalignments. For hSPEPS$^{CA\text{-}CO}$, the optimal rf-field strength is

observed in the expected area – approximately ν_R (labeled with the green arrow), while for hTROP, the experimentally optimal rf-field strength deviated by 23 kHz from the proposed value of 75 kHz.[7]

Figure S9B shows 1D (HCACON)H (black) and (HCOCAN)H (blue) signals as a function of hSPEPS$^{CA\text{-}CO}$ mixing time. The maximal CA-CO and CO-CA transfers are achieved with 1.16 ms mixing. For hTROP, the optimal mixing time is fixed and equals 1.8 ms at 55 kHz MAS.

Figure S9D-E compares 1D (HCACON)H (D) and (HCOCAN)H spectra, obtained with hSPEPS$^{CA\text{-}CO}$ (black) and hTROP (red) sequences. At a 600 MHz spectrometer, hSPEPS$^{CA\text{-}CO}$ yields higher transfer efficiency for CA-CO transfers compared to hTROP (D). For CO-CA transfers, both sequences provide similar transfer efficiency (Figure S9E) in 1D.

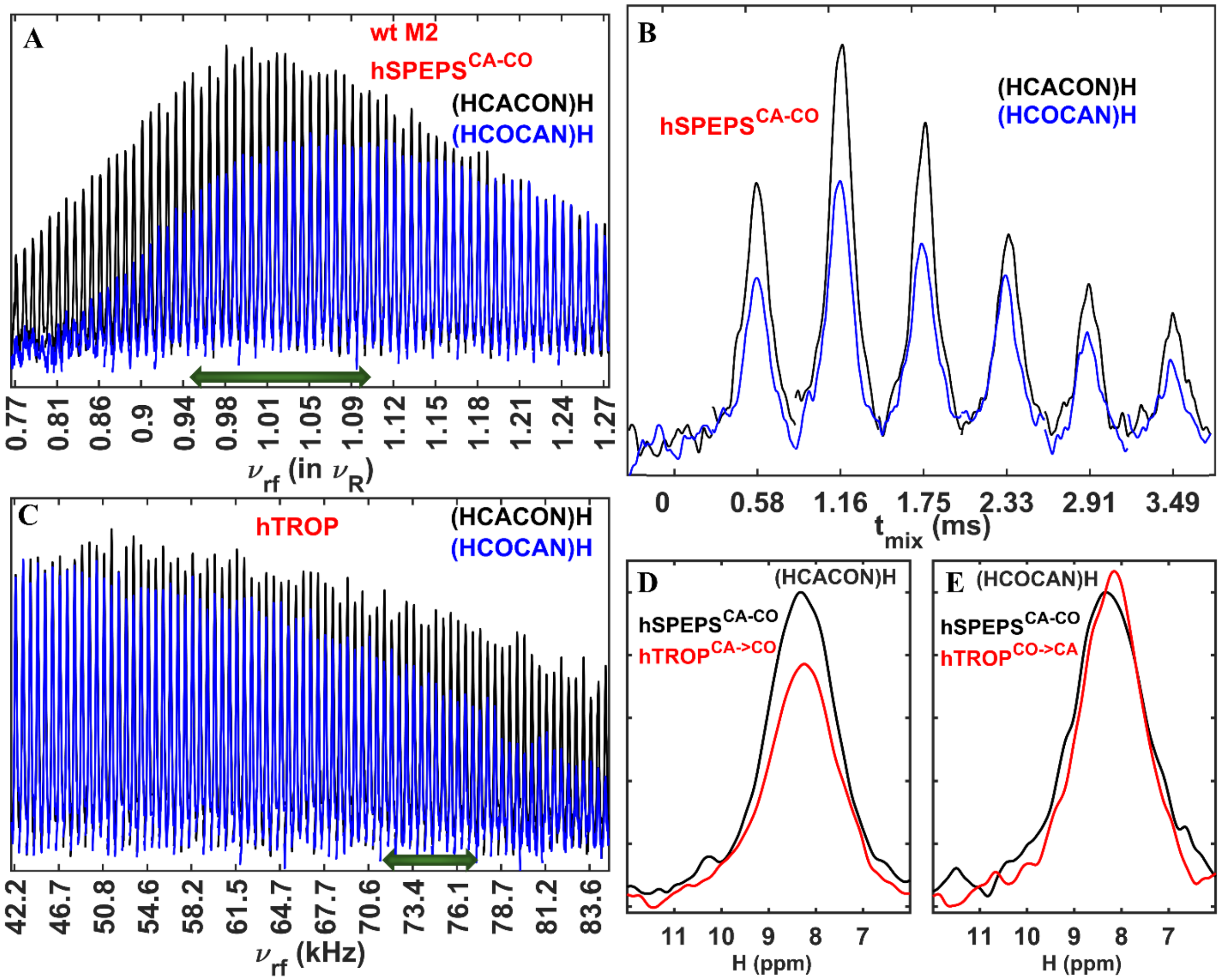

Figure S9 1D (HCACON)H and (HCOCAN)H optimizations for hSPEPS$^{CA\text{-}CO}$ and hTROP sequences. (A)-(B) hSPEPS$^{CA\text{-}CO}$ rf-field strength (A) and mixing time (B) optimization for CA-CO (black) and CO-CA (blue) transfers. (C) hTROP rf-field optimization for CA→CO (black) and CO→CA (blue) transfers. Different shaped pulse profiles were used for CA→CO and CO→CA transfers, which were developed by Blahut et al.[7] for 600 MHz spectrometer and 55 kHz MAS. The shaped pulse profiles were downloaded from: https://optimal-nmr.net/sequences.html. The mixing time was set to 1.8 ms according to the article by Blahut et al.[7] Comparison of (HCACON)H (D) and (HCOCAN)H (E) spectra with hSPEPS$^{CA\text{-}CO}$ (black) and hTROP (red) sequences. The spectra were acquired at a 600 MHz spectrometer with 55 kHz MAS using ^{13}C, ^{15}N-labelled WT M2, containing Cu^{2+} ethylenediaminetetraacetic acid to accelerate the acquisition. Further details of the experiments are presented below.

Figure S10A-B displays 2D (H)CC spectra, where CO-CA cross peaks were excited with hSPEPS$^{CA\text{-}CO}$ (black-negative and cyan-positive) and hTROP (red) sequences. The hSPEPS$^{CA\text{-}CO}$ sequence can simultaneously excite CA→CO and CO→CA transfers (black cross peaks in Figure S10A), while with hTROP, expectedly, only CO→CA transfers (red) are observed. In Figure S10C, four slices from the 2D (H)CACO spectra (Figure S10B) are shown and quantitatively compared with the diagonal peaks from a 2D (H)CC experiment with zero mixing time (gray). In general, hSPEPS performs better than hTROP under these experimental conditions. Four selected slices from these spectra are shown in Figure S10C.

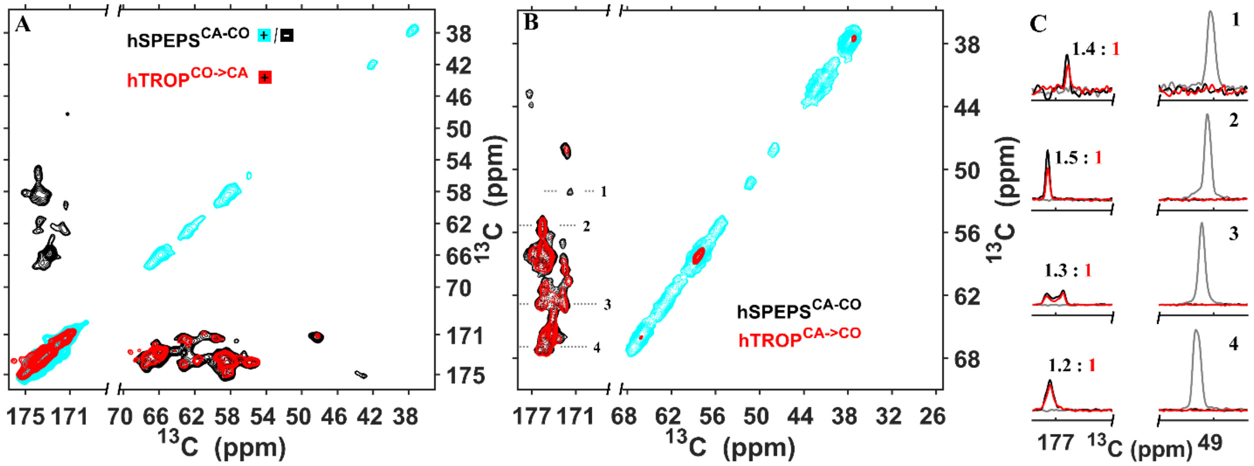

Figure S10 2D (H)CC spectra with hSPEPS$^{CA\text{-}CO}$ (black, 1.16 ms mixing) and hTROP (red, 1.8 ms mixing) sequences for CA-CO (A) and CO-CA (B) transfers. The spectra were acquired on a 600 MHz spectrometer with 55 kHz MAS using ^{13}C, ^{15}N-labelled WT M2, containing Cu^{2+} ethylenediaminetetraacetic acid to accelerate the acquisition. The rf-field strengths for hSPEPS$^{CA\text{-}CO}$ and hTROP sequences were set to 58 kHz and 60 kHz, respectively. (A) The carrier frequency was set at 173.7 ppm for ^{1}H→^{13}C CP transfers. Before the hSPEPS$^{CA\text{-}CO}$ sequence, it was shifted to 113.7 ppm. For the hTROP sequence, it remained at 173.7 ppm. (B) The carrier frequency was set at 53.7 ppm for ^{1}H→^{13}C CP transfers. Before the hSPEPS$^{CA\text{-}CO}$ sequence, it was shifted to 113.7 ppm. For the hTROP sequence, it remained at 53.7 ppm. (C) Four slices from 2D spectra, shown in Figure S5B (black and red spectra). Gray lines are the diagonal slices from the 2D (H)CC experiment with zero mixing time. The ratios show the improvement relative to the TROP sequence.

Figure S11 displays ^{13}C-^{15}N projections from 3D (H)CO(CA)NH (A) and 3D (H)CA(CO)NH (B), recorded at a 600 MHz and at a 1200 MHz, respectively.

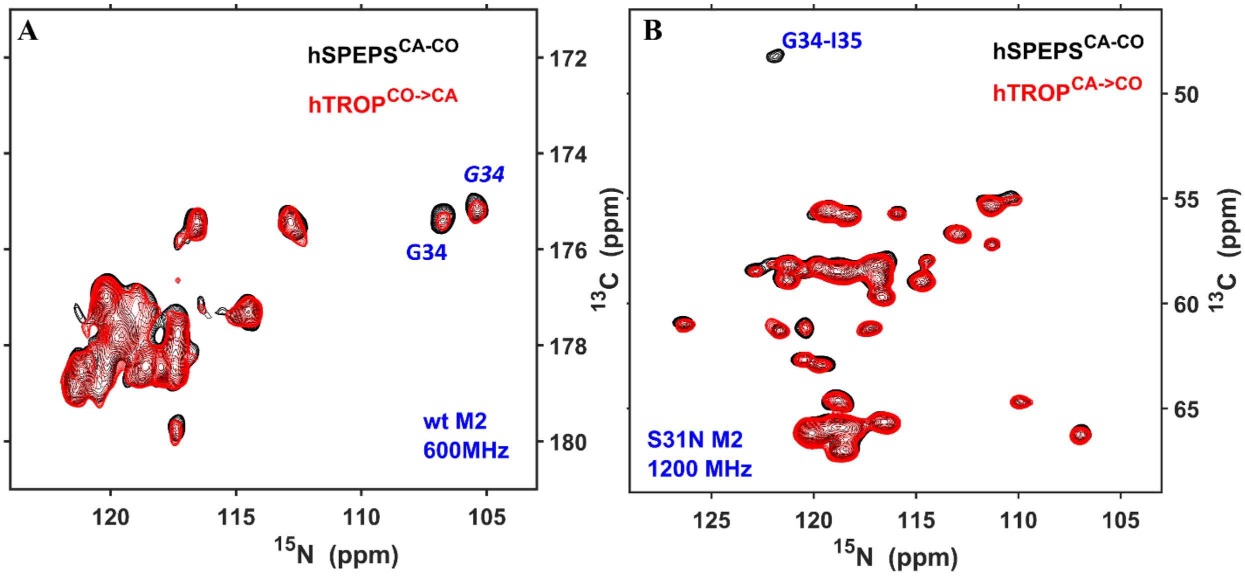

Figure S11 ^{13}C-^{15}N projections from 3D (H)CO(CA)NH (A) and 3D (H)CO(CA)NH. (A) Black represents hSPEPS$^{CA\text{-}CO}$ (1.16 ms mixing) and red represents hTROP$^{CO\rightarrow CA}$ (1.8 ms mixing) for CO→CA transfers. During hSPEPS$^{CA\text{-}CO}$ and hTROP$^{CO\rightarrow CA}$ sequences, the carrier frequency was set to 113.7 ppm and 173.7 ppm, respectively. The data were recorded with the WT M2 sample, at a 600 MHz external magnetic field and 55 kHz MAS. (B) Black represents hSPEPS$^{CA\text{-}CO}$ (1.16 ms mixing) and red represents hTROP$^{CA\rightarrow CO}$ (1.8 ms mixing) for CA→CO transfers. During hSPEPS$^{CA\text{-}CO}$ and hTROP$^{CA\rightarrow CO}$ sequences, the carrier frequency was set to 113.7 ppm and 53.7 ppm, respectively. The data were recorded with the S31N M2 sample, at a 1200 MHz external magnetic field and 55 kHz MAS. Different hTROP shaped pulse profiles were used for CO→CA and CA→CO transfers, which were developed by Blahut et al.[7] for the 600 MHz spectrometer and 55 kHz MAS (CO→CA) and for the 1200 MHz spectrometer and 55 kHz MAS (CA→CO). The shaped pulse profiles were downloaded from: https://optimal-nmr.net/sequences.html. Further details of the experiments are presented below.

EXPERIMENTAL METHODS

Simulations

SPEPS simulations were performed using in-house MATLAB scripts to compute the numerical solution of the equation of motion.[13] For simulations in Figure 1C-D in the main text

we used a three spin system (I_3, three carbon spins). For the CA-CB simulations (Figure 1C in the main text), the homonuclear dipolar coupling values between CO-CA, CO-CB and CA-CB were 2.13 kHz, 0.48 kHz and 15 kHz, respectively. The CA-CB dipolar coupling was increased sevenfold to enhance and better illustrate the transfer during the first few rotor periods. For CA-CO simulations (Figure 1D in the main text), the homonuclear dipolar coupling values between CO-CA, CO-CB and CA-CB were 15 kHz, 0.48 kHz, and 2.12 kHz, respectively. Similarly, the CO-CA dipolar coupling was increased sevenfold to enhance the rate of transfer for illustrative purposes. In all simulations, the isotropic chemical shifts and chemical shift anisotropy (in ppm) were [177;53;19] and [110;70;60], respectively.

Sample Preparation

Influenza A M2 samples

Influenza A wild type M2 and S31N M2 proteins, residues 18-60, were prepared according to the protocols in the references [[14,15]]. The samples were packed into Bruker 1.3 mm rotors via centrifugation.

Bacterial rhomboid protease GlpG

[^{13}C,^{15}N]-labeled full-length (residues 1-276) bacterial rhomboid protease GlpG was expressed and purified as published previously.[16] The purified protein was reconstituted into a perdeuterated *E. coli* total lipid extract (produced in our lab following the protocol published by Bligh and Dyer in 1959[17]). In brief, the deuterated lipid extract was solubilized with 3% n-decyl β-maltoside (DM, GLYCON Biochemicals, Germany) detergent buffer and mixed with the purified GlpG at a protein-to-lipid ratio of 1:1 w/w (~ 1:30 mol/mol). Detergent removal was carried out by dialyzing at 100 times dilution against dialysis buffer [50 mM Tris-HCl, 300 mM NaCl, 1 mM MgCl, 1.4 mM β-mercaptoethanol, pH 7.4]. Biobeads (Biorad) were added to the dialysis buffer to facilitate detergent removal. The dialysis process lasted for 10 days with

multiple buffer exchanges (at least every second day). A rotor packing device,[18] crafted in our institute (Leibniz-Forschungsinstitut für Molekulare Pharmakologie), was used to fill up a 0.7 mm rotor (Bruker). For this purpose, 0.45 mg of the proteoliposomes and 250 µL of dialysis buffer saturated with sodium trimethylsilylpropanesulfonate (DSS) for referencing and temperature control were loaded into the funnel of the apparatus, which was later assembled and centrifuged (12 h, 4 ^{o}C, at 71,000 g).

Solid State NMR Spectroscopy

hSPEPS sequence set up

For hSPEPS$^{CA\text{-}CB}$, the optimal rf-field strength is $1.25\nu_R$. The required rf-field power can be calculated according to the calibrated ^{13}C 90°-pulse (e.g., 1D (H)C followed by a 90°-pulse on the carbon channel). The optimal mixing time for direct carbon-carbon excitations is around 1 ms. During hSPEPS$^{CA\text{-}CB}$, the ^{13}C CF should be set in the middle of carbon aliphatic region (~40 ppm).

For hSPEPS$^{CA\text{-}CO}$, the optimal rf-field strength varies around ν_R, depending on the MAS rate and the external magnetic field. The optimal rf-field power can be determined experimentally by recording a series of 1D (HCACON)H and 1D (HCOCAN)H spectra as a function of rf-field power. The optimal mixing time for CA-CO excitations is around 1 ms. During hSPEPS$^{CA\text{-}CO}$, the ^{13}C CF should be set midway between the CA and CO regions of the spectrum (~113 ppm).

Artifacts in multi-dimensional experiments

All 2D and 3D experiments include a pair of 90°-pulses before the hSPEPS element. During recording multi-dimensional signals, the phase of the second 90°-pulse is shifted by π every even scan, which results to the inversion of the one of the transverse components in these

cases. The F_1 indirect dimension, recorded with the Echo/Anti-Echo mode, is converted into the STATES-TPPI mode[19,20] after applying the Echo/Anti-Echo → STATES/ STATES-TPPI conversion script.[5] In this case, the artifacts are placed at the edge of the spectrum. An additional solution is to place the CF in a spot where the peaks of interest should not be observed.

We also suggest an optional filter after H to CA CP that removes the CA signal in (H)CO(CA)NH experiments (or CO signal in (H)CAcoNH experiments) and therefore reduces the requirements of the phase cycling. It also improves the 1D estimate of the true performance of each sequence.

We have also found that there is sometimes an unwanted transfer pathway that can lead to signal cancellation and negative ‘artifact’ peaks. In this case, there is also unwanted signal detected with zero hSPEPS$^{CA\text{-}CO}$ mixing. This unwanted signal path results from, e.g., undesired ^{13}CO-^{15}N transfers during the ^{13}CA-^{15}N SPEPS transfer step (in (H)CO(CA)NH experiments). The sign to the signal was opposite to the intended signal, as expected after DQ CO-CA transfer. For such cases, we used the modified (H)CO(CA)NH sequence (m(H)CO(CA)NH, below) with an additional block of soft pulses between hSPEPS$^{CA\text{-}CO}$ and SPEPS elements to remove the unwanted signal.

Sequences and experimental parameters

Figures S12 displays 2D (H)CC with DREAM, hSEPS$^{CA\text{-}CB}$, hTROP and hSEPS$^{CA\text{-}CO}$ transfer sequences. Figure S13 displays 3D (H)CA(CO)NH and 3D (H)CO(CA)NH pulse sequences with hTROP and hSEPS$^{CA\text{-}CO}$ transfer elements. Figure S14 displays 3D (H)CB(CA)NH with hSEPS$^{CA\text{-}CB}$ Figures S15 and S16 display (HCO)CA(CO)NH and (HCA)CB(CA)NH with J-based transfer elements.[21]

Common experimental conditions: In all proton-detected experiments (including hSPEPS, hTROP and j-based sequences), SPEPS[5] was applied for $^{13}C \rightarrow ^{15}N$ transfers, while for $^{1}H \rightarrow ^{13}C$ and $^{15}N \rightarrow ^{1}H$ transfers, ramped CP sequences[6] were applied. The optimal conditions for ^{13}CA-^{15}N and ^{13}CO-^{15}N SPEPS were obtained from optimization of 1D (HCAN)H and 1D (HCON)H signals. For ^{1}H decoupling, the SW$_f$-TPPM sequence[22] was applied in all ^{13}C and ^{15}N acquisition periods, as well as during J-transfer elements.

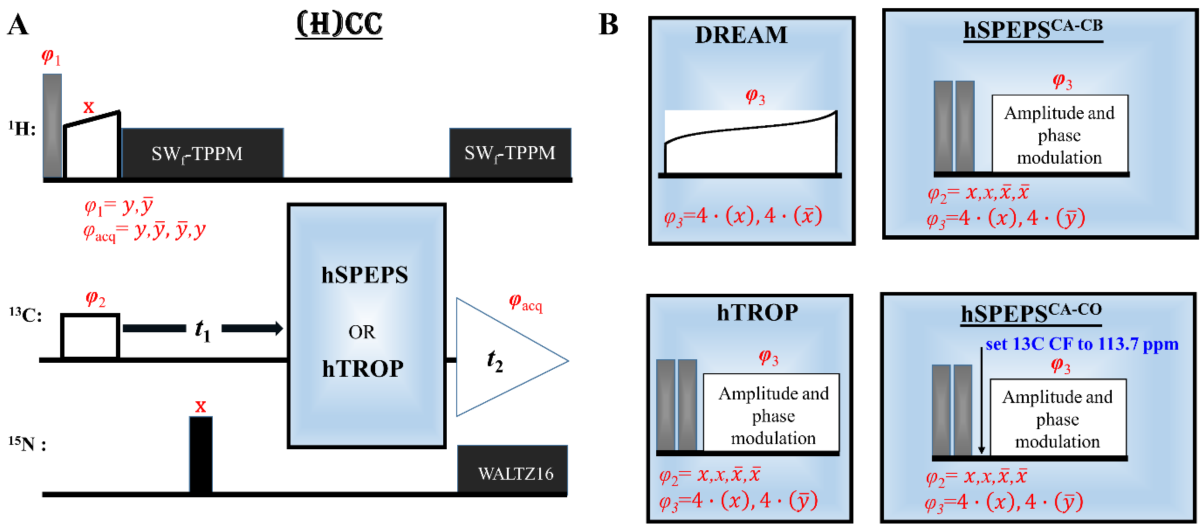

Figure S12 2D (H)CC pulse sequence with either DREAM, hTROP or hSPEPS recoupling sequences. $\pi/2$-pulses are indicated by light, π-pulses by dark grey rectangles. All phase cycling is shown in figure. The ramped CP transfers from proton to carbon are indicated with constant power on the carbon channel and a ramp in power on the proton channel. During the indirect dimension (t_1) and acquisition (t_2), SW$_f$-TPPM decoupling[22] is applied. A single π-pulse in the middle of t_1 and t_2 decouples carbon-nitrogen interactions. DREAM: a TAN amplitude modulated shape was used.[10,12] hTROP: the hTROP shaped pulse lists were taken from: https://optimal-nmr.net/sequences.html, and applied without any modifications. hSPEPS: the hSPEPS elements are displayed in Figure 1 in the main text. During hSEPS$^{CA\text{-}CB}$, the ^{13}C CF should be set in the middle of carbon aliphatic region (~42 ppm). During hSPEPS$^{CA\text{-}CO}$, ^{13}C CF should be set in the middle between the Ca and CO groups (~113 ppm).

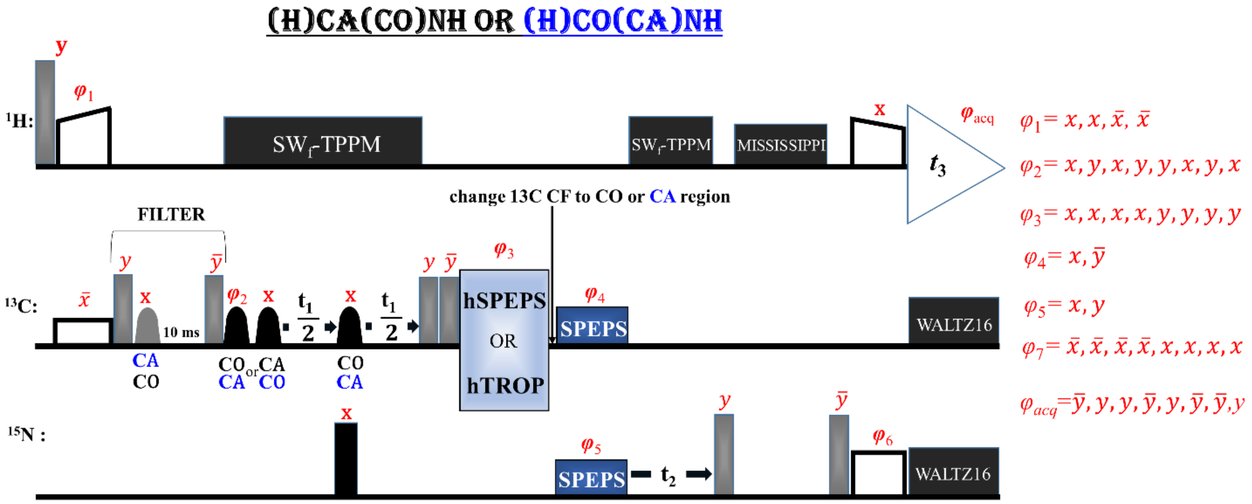

Figure S13 3D (H)CA(CO)NH or 3D (H)CO(CA)NH with either hSPEPS$^{CA\text{-}CO}$ or hTROP recoupling elements. π/2-pulses are indicated by light rectangles and π-pulses by black rectangles. Ramped CP is used for proton to carbon transfer and from nitrogen to proton transfers. For $^{13}C \rightarrow {}^{15}N$ transfers, SPEPS was implemented as a shaped pulse list with constant amplitude modulation.[5] During the indirect dimension (t_1) and acquisition (t_2), SW$_f$-TPPM decoupling[22] is applied. π-pulses in the middle of t_1 and t_2 are used to decouble carbon-nitrogen interactions. REBURP π (black) and π/2 (gray) soft selective pulses[23] are applied on carbon channel to select either ^{13}CA or ^{13}CO spins. During acquisition, WALTZ16 decoupling[24] was applied on nitrogen and carbon channels. FILTER is an optional block that eliminates the undesired initial CO or CA intensities for (H)CA(CO)NH or (H)CO(CA)NH experiments, respectively.

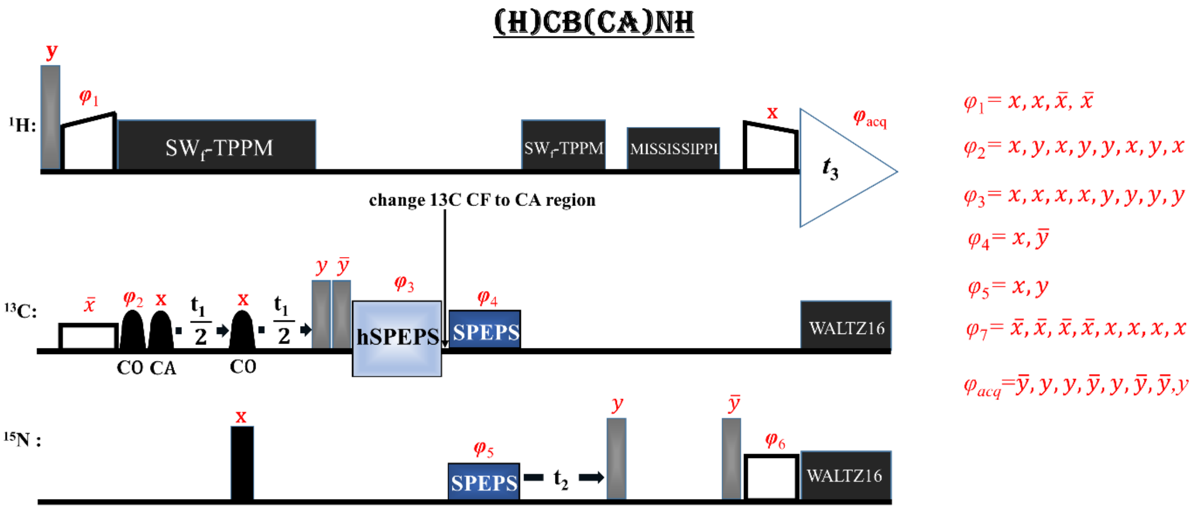

Figure S14 3D (H)CB(CA)NH with hSPEPS$^{CA\text{-}CB}$ recoupling element. π/2-pulses are indicated by light rectangles and π-pulses by black rectangles. Ramped CP is used for proton to carbon transfer and from nitrogen to proton transfers. For $^{13}C \rightarrow {}^{15}N$ transfers, SPEPS was implemented as a shaped pulse list with constant amplitude modulation.[5] During the indirect dimension (t_1) and acquisition (t_2), SW$_f$-TPPM decoupling[22] is applied. π-pulses in the middle of t_1 and t_2 are used to decouble carbon-nitrogen interactions. REBURP soft selective pulses[23] are applied on carbon channel to select ^{13}CA spins. During acquisition, WALTZ16 decoupling[24] was applied on nitrogen and carbon channels.

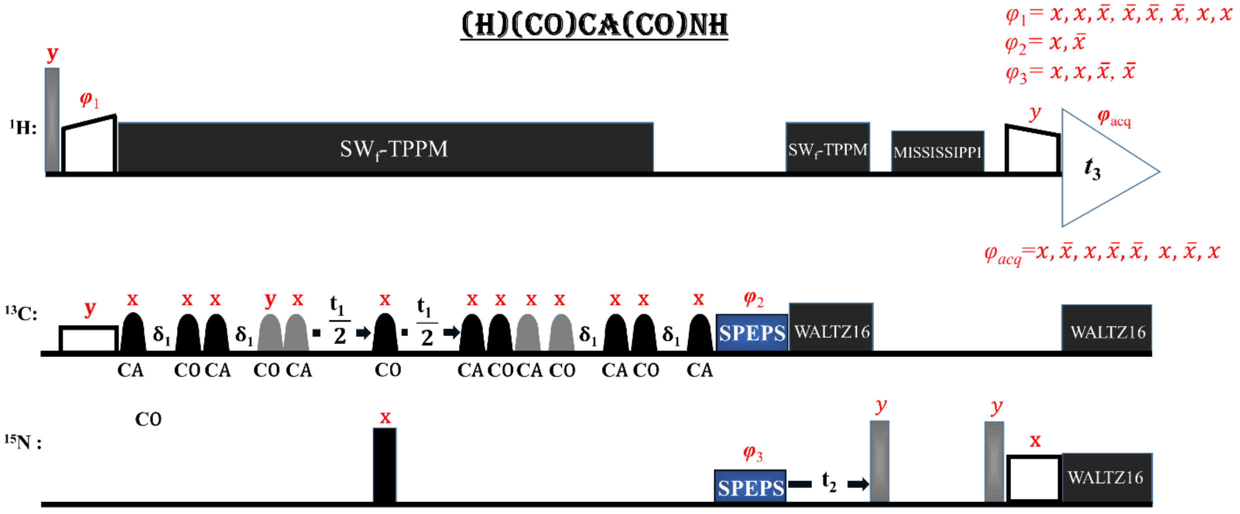

Figure S15 3D (H)(CO)CA(CO)NH with J-based recoupling element. π/2-pulses are indicated by light rectangles and π-pulses by black rectangles. Ramped CP is used for proton to carbon transfer and from nitrogen to proton

transfers. For $^{13}C \rightarrow ^{15}N$ transfers, SPEPS was implemented as a shaped pulse list with constant amplitude modulation.[5] During the indirect dimension (t_1) and acquisition (t_2), SW_f-TPPM decoupling[22] is applied. π-pulses in the middle of t_1 and t_2 are used to decouble carbon-nitrogen interactions. REBURP π (black) and π/2 (gray) soft selective pulses[23] are applied on carbon channel to select either ^{13}CA or ^{13}CO spins. During acquisition, WALTZ16 decoupling[24] was applied on nitrogen and carbon channels.

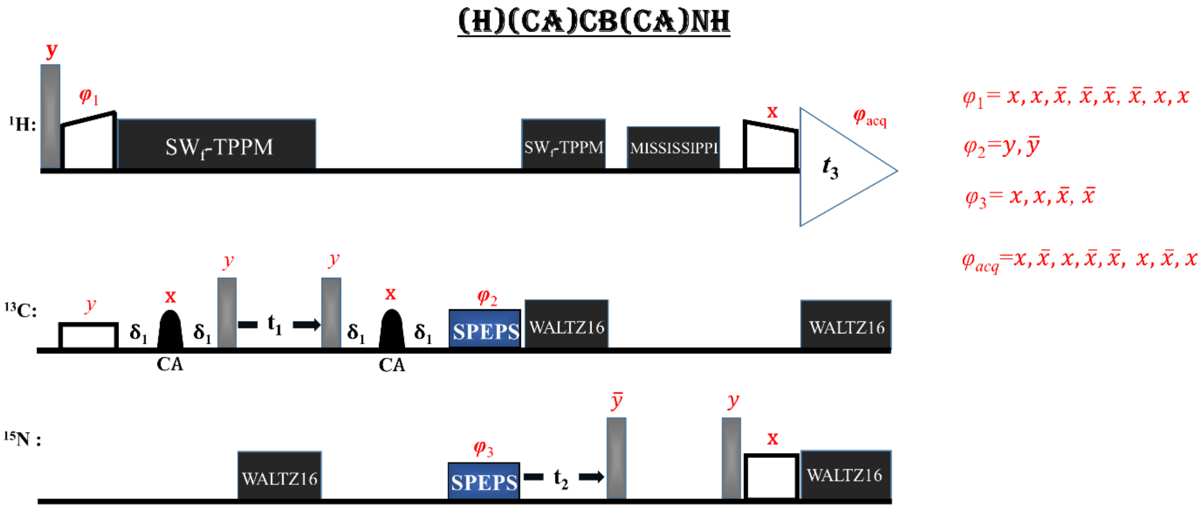

Figure S16 3D (H)(CA)CB(CA)NH with J-based recoupling element. π/2-pulses are indicated by light rectangles and π-pulses by black rectangles. Ramped CP is used for proton to carbon transfer and from nitrogen to proton transfers. For $^{13}C \rightarrow ^{15}N$ transfers, SPEPS was implemented as a shaped pulse list with constant amplitude modulation.[5] During the indirect dimension (t_1) and acquisition (t_2), SW_f-TPPM decoupling[22] is applied. π-pulses in the middle of t_1 and t_2 are used to decouble carbon-nitrogen interactions. REBURP soft selective pulses[23] are applied on carbon channel to select ^{13}CA spins. During acquisition, WALTZ16 decoupling[24] was applied on nitrogen and carbon channels.

600 MHz: Experiments acquired at 600 MHz utilized a Bruker Avance III HD spectrometer operating at 14.1 T (600 MHz ^{1}H frequency) using a DVT600W2 BL1.3 mm HXY probe. The experiments were performed at 55 kHz MAS, and the temperature of the nitrogen cooling gas set to 245 K with 1000 to 1300 liters per hour. For decoupling of the heteronuclear dipolar interactions SW_f-TPPM[22] or WALTZ-16[24] were used on the proton channel, and WALTZ-16[24]

was used on heteronuclear channels. MISSISSIPPI[25] water suppression was applied for proton detected experiments.

In Figure S17 (**the data are shown in Figure S7**), below: SW – spectral width; TD – the number of points in the FID; IN_F – an increment time; AQ – the acquisition time. 2 Dummy Scans were used.

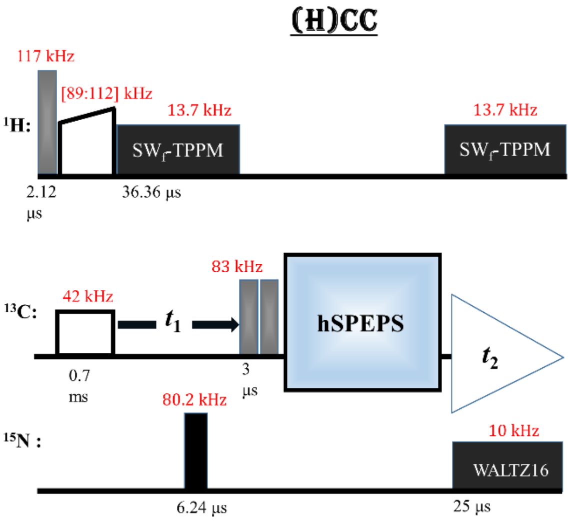

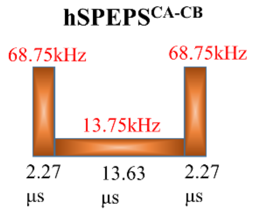

(H)CC	^{13}C	^{13}C
TD	3324	1000
SW(ppm)	553.1672	320
IN_F(µs)		20.7438
AQ(s)	0.019944	0.0103719

CF=42 ppm | Number Scans: 40 | Recycle delay: 0.3 s
Total Time of the experiment: 3.71 hours |

Figure S17 The 2D (H)CC sequence and the experimental parameters used for measurement on wt M2 with hSPEPS$^{CA\text{-}CB}$ element. Red numbers represent rf-field strength in kHz. The width of the hard pulses are in µs, while total duration of CP and decoupling are in ms. For H→C transfers CP with [80%:100%] ramp is applied on proton channel. hSPEPS$^{CA\text{-}CB}$, rf-field strengths and the pulse widths are shown with respect to 55 kHz MAS.

In Figure S18 (**the data are shown in Figure S8**), below: SW – spectral width; TD – the number of points in the FID; IN_F – an increment time; AQ – the acquisition time. 2 Dummy Scans were used.

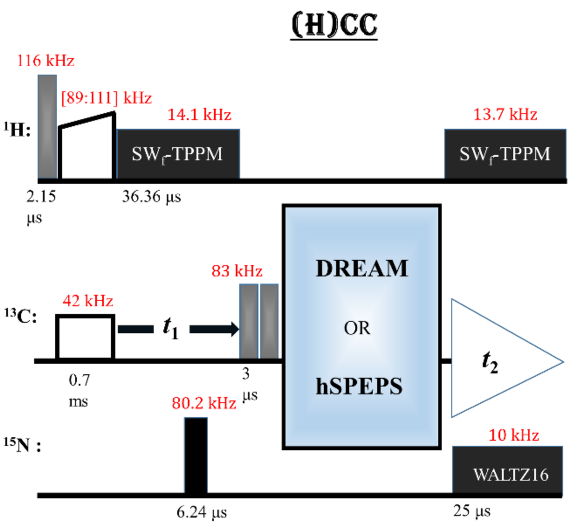

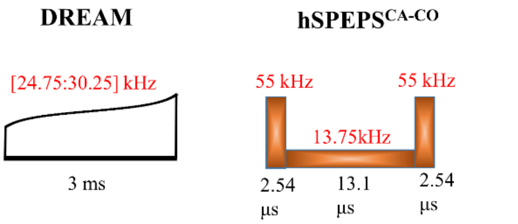

(H)CC	^{13}C	^{13}C
TD	3324	1024
SW(ppm)	553.1672	320
IN_F(µs)		20.7438
AQ(s)	0.019944	0.0106208

CF=42 ppm | Number Scans: 48 | Recycle delay: 0.3 s
Total Time of the experiment: 4.56 hours |

Figure S18 The 2D (H)CC sequence and the experimental parameters used for measurement on wt M2 with hSPEPS$^{CA\text{-}CB}$ element. Red numbers represent rf-field strength in kHz. The width of the hard pulses are in µs, while total duration of CP and decoupling are in ms. For H→C transfers CP with [80%:100%] ramp is applied on proton channel. hSPEPS$^{CA\text{-}CB}$, rf-field strengths and the pulse widths are shown with respect to 55 kHz MAS.

In Figure S19 (**the data are shown in Figure S10**), below: SW – spectral width; TD – the number of points in the FID; IN_F – an increment time; AQ – the acquisition time. 2 Dummy Scans were used.

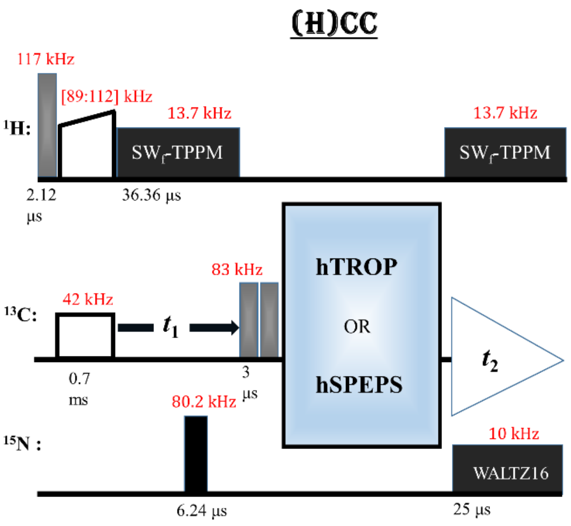

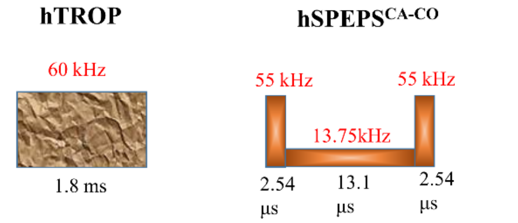

(H)CC	^{13}C	^{13}C
TD	3324	1000
SW(ppm)	553.1672	320
IN_F(µs)		20.7438
AQ(s)	0.019944	0.0103719

CF=53.7 ppm | Number Scans: 64 | Recycle delay: 0.3 s
Total Time of the experiment: 5.94 hours |

Figure S19 The 2D (H)CC sequence and the experimental parameters used for measurement on wt M2 with hTROP and hSPEPS$^{CA\text{-}CB}$ elements. Red numbers represent rf-field strength in kHz. The width of the hard pulses are in µs, while total duration of CP and decoupling are in ms. For H→C transfers CP with [80%:100%] ramp is applied on proton channel. For hTROP and hSPEPS$^{CA\text{-}CB}$, rf-field strengths and the pulse widths are shown with respect to 55 kHz MAS.

Table S1 summarizes the experimental parameters of recoupling elements used for the 3D (H)CA(CO)NH and (H)CO(CA)NH spectrum of wt M2 (**Figures 2A in the main text and S11A**). Additional parameters are shown in Figure S20.

**3D (H)CA(CO)NH** / **3D (H)CO(CA)NH**		hTROP	hSPEPS
^{1}H→^{13}CA	$\nu_{rf}(^{1}H)$	linear ramp: [80%:100%] [87:109] (kHz)	linear ramp: [80%:100%] [87:109] (kHz)
	$\nu_{rf}(^{13}C)$	42 kHz	42 kHz
	t_{mix}	0.6 ms / **2 ms**	0.6 ms / **2ms**
**^{13}CA→^{13}CO** / **^{13}CO→^{13}CA**	$\nu_{rf}(^{13}C)$	**56.9 kHz / 51.4 kHz**	**58.2 kHz / 57 kHz**
	t_{mix}	**1.8 ms / 1.8 ms**	**1.16 ms / 1.16 ms**
^{13}CA→^{15}N	$\nu_{rf}(^{15}N)$	42 kHz	42 kHz
	$\nu_{rf}(^{13}C)$	13.6 kHz	13.6 kHz
	t_{mix}	2.3272 ms<br>2.90909 ms	2.3272ms<br>2.90909 ms
^{15}N→^{1}H	$\nu_{rf}(^{1}H)$	linear ramp: [100%:80%] [111:89] (kHz)	linear ramp: [100%:80%] [111:89] (kHz)
	$\nu_{rf}(^{15}N)$	43 kHz	43 kHz
	t_{mix}	0.5 ms	0.5 ms

Table S1 The shapes, nominal rf-field strengths and transfer times for recoupling elements as implemented for ^{1}H-^{13}CA, ^{13}CA-^{15}N and ^{15}N-^{1}H transfers at 55 kHz MAS. For ramped CP, rectanglular brackets indicate the minimal

and maximal rf-field of ramped rf-field strengths. For hTROP, the hTROP shape files were downloaded from https://optimal-nmr.net/sequences.html and used without any modification. In the table the maximal hTROP rf-field strengths and hSPEPS are shown. For all elements, rf-field strengths values and mixing times indicate the experimentally determined optimum.

In Figure S20 below: SW – spectral width; TD – the number of points in the FID; IN_F – an increment time; AQ – the acquisition time. 2 Dummy Scans were used.

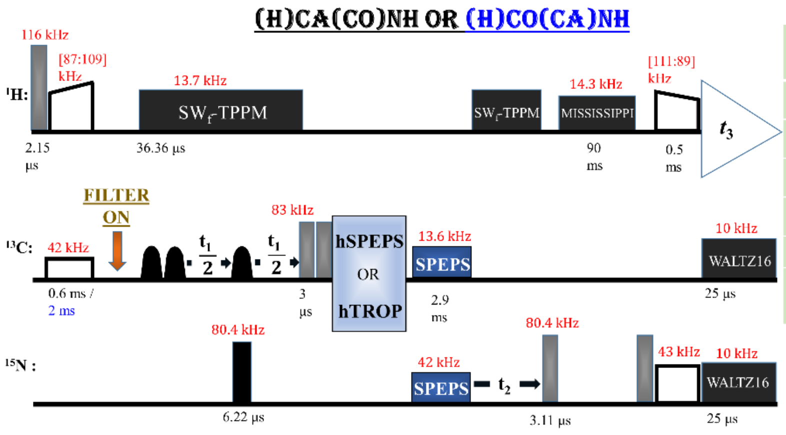

(H)CA(CO)NH<br>(H)CO(CA)NH	F3	F2	F1
	^{1}H	^{15}N	^{13}C
TD	1386	110	120
SW(ppm)	46.3664	50	50
IN_F(µs)		329.42	132.76
AQ(s)	0.024948	0.0181182	0.0078328
CF	4.7	118	50<br>168

Number Scans: 16
Recycle delay: 0.3 s
Total Time of the experiment: 27.17 hours

Figure S20 The 3D (H)CA(CO)NH (^{13}C CF set to 53. 7 ppm) and (H)CO(CA)NH (^{13}C CF set to 168 ppm) sequence and the experimental parameters (Figures 2A in the main text and Figure S11A). Red numbers represent rf-field power in kHz. The hard pulse durationss are shown in µs, while total duration of CP and decoupling are in ms. For $^{1}H\rightarrow^{13}C$ transfers, CP with [80%:100%] ramp was applied on the proton channel. For $^{15}N\rightarrow^{1}H$ transfers, CP with [100%:80%] ramp was applied on the proton channel. For ramped CP, rectanglular brackets indicate the minimal and maximal rf-field of ramped rf-field strengths. The details of $^{1}H\rightarrow^{13}CA$, $^{13}CA\rightarrow^{15}N$ and $^{15}N\rightarrow^{1}H$ transfers (the shapes, the rf-field strengths and mixing times) are summarized in Tables S1. The optional block, FILTER ON (shown Figure S13), was applied for elimenating the undesired initial CO and CA intensities for (H)CA(CO)NH and (H)CO(CA)NH experiments, respectively.

Table S2 summarizes the experimental parameters of recoupling elements used for the 3D (H)CB(CA)NH (with hSPEPS) and (HCA)CB(CA)NH (with J-based) spectrum of wt M2 (**Figures 2C in the main text**). Additional parameters are shown in Figure S21 for 3D

(H)CB(CA)NH. For 3D (HCA)CB(CA)NH experiments, exactly the same NS and the total time were used as for 3D (H)CB(CA)NH experiment.

**3D (H)CB(CA)NH** **3D (HCA)CB(CA)NH**		J-based	hSPEPS
$^{1}H \rightarrow ^{13}C$	$\nu_{rf}(^{1}H)$	linear ramp: [80%:100%] [90:112] (kHz)	linear ramp: [80%:100%] [90:112] (kHz)
	$\nu_{rf}(^{13}C)$	42 kHz	42 kHz
	t_{mix}	0.6 ms	0.6 ms
**$^{13}CB \rightarrow ^{13}CA$**	$\nu_{rf}(^{13}C)$	**Train of Soft Pulses**	**68.75 kHz**
	t_{mix}	**13.79 ms**	**1.16 ms**
$^{13}CA \rightarrow ^{15}N$	$\nu_{rf}(^{15}N)$	39.19 kHz	39.19 kHz
	$\nu_{rf}(^{13}C)$	13.75 kHz	13.75 kHz
	t_{mix}	2.90909 ms	2.90909 ms
$^{15}N \rightarrow ^{1}H$	$\nu_{rf}(^{1}H)$	linear ramp: [100%:80%] [114:91] (kHz)	linear ramp: [100%:80%] [114:91] (kHz)
	$\nu_{rf}(^{15}N)$	42 kHz	42 kHz
	t_{mix}	0.5 ms	0.5 ms

Table S2 The shapes, nominal rf-field strengths and transfer times for recoupling elements as implemented for ^{1}H-^{13}CA, ^{13}CA-^{15}N and ^{15}N-^{1}H transfers at 55 kHz MAS. For ramped CP, rectanglular brackets indicate the minimal and maximal rf-field of ramped rf-field strengths. In the table the maximal hSPEPS are shown. For J-based, a train of soft pulses has been applied as depicted in Figure S15. For all elements, rf-field strengths values and mixing times indicate the experimentally determined optimum.

In Figure S21 below: SW – spectral width; TD – the number of points in the FID; IN_F – an increment time; AQ – the acquisition time. 2 Dummy Scans were used.

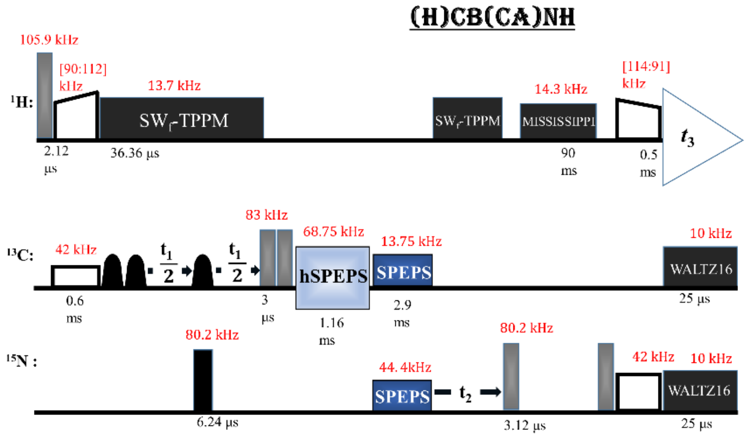

(H)CB(CA)NH	F3	F2	F1
	^{1}H	^{15}N	^{13}C
TD	1386	110	150
SW(ppm)	46.3664	50	56
IN_F(µs)		329.42	118.54
AQ(s)	0.024948	0.0164711	0.0073017
CF	4.7	118	39.7

Number Scans: 16
Recycle delay: 0.3 s
Total Time of the experiment: 1.35 days

Figure S21 The 3D (H)CB(CA)NH sequence and the experimental parameters (Figures 2C). Red numbers represent rf-field power in kHz. The hard pulse durationss are shown in µs, while total duration of CP and decoupling are in ms. For $^{1}H \rightarrow ^{13}C$ transfers, CP with [80%:100%] ramp was applied on the proton channel. For $^{15}N \rightarrow ^{1}H$ transfers, CP with [100%:80%] ramp was applied on the proton channel. For ramped CP, rectanglular brackets indicate the minimal and maximal rf-field of ramped rf-field strengths. The details of $^{1}H \rightarrow ^{13}CA$, $^{13}CA \rightarrow ^{15}N$ and $^{15}N \rightarrow ^{1}H$ transfers (the shapes, the rf-field strengths and mixing times) are summarized in Tables S2.

1200 MHz, 1.3 mm probe: Experiments acquired at a 1200 MHz on a Bruker Avance NEO spectrometer operating at 28.18 T (1200 MHz ^{1}H frequency) using a 1.3 mm HCN probe. The experiments were performed at 55.555 kHz MAS, the temperature of the nitrogen cooling gas set to 245 K using 1000 liters per hour of flow. For decoupling of the heteronuclear dipolar interactions and water suppression SW_f-TPPM,[22] WALTZ-16[24] and MISSISSIPPI[25] were applied. In the Figure below: SW – spectral width; TD – the size of FID; IN_F – an increment time; AQ – the acquisition time. 2 Dummy Scans were used.

Table S3 summarizes the experimental parameters of recoupling elements used for the 3D (H)CA(CO)NH spectrum of S31N M2 (**Figure S11B**). Additional parameters are shown in Figure S22.

**3D (H)CA(CO)NH**		hTROP	hSPEPS

^{1}H→^{13}CA	ν_{rf}(^{1}H)	linear ramp: [80%:100%] [88:110] (kHz)	linear ramp: [80%:100%] [88:110] (kHz)
	ν_{rf}(^{13}C)	42 kHz	42 kHz
	t_{mix}	0.6 ms	0.6 ms
**^{13}CA→^{13}CO**	ν_{rf}(^{13}C)	**70.7 kHz**	**47.5 kHz**
	t_{mix}	**1.8 ms**	**1.16 ms**
^{13}CA→^{15}N	ν_{rf}(^{15}N)	40.5 kHz	40.5 kHz
	ν_{rf}(^{13}C)	12.2 kHz	12.2 kHz
	t_{mix}	2.91 ms	2.91 ms
^{15}N→^{1}H	ν_{rf}(^{1}H)	linear ramp: [100%:80%] [112:89] (kHz)	linear ramp: [100%:80%] [112:89] (kHz)
	ν_{rf}(^{15}N)	42 kHz	42 kHz
	t_{mix}	0.5 ms	0.5 ms

Table S3 The shapes, nominal rf-field strengths and transfer times for recoupling elements as implemented for ^{1}H-^{13}CA, ^{13}CA-^{15}N and ^{15}N-^{1}H transfers at 55 kHz MAS. For ramped CP, rectanglular brackets indicate the minimal and maximal rf-field of ramped rf-field strengths. For hTROP, the hTROP shape files were downloaded from https://optimal-nmr.net/sequences.html and used without any modification. In the table the maximal hTROP rf-field strengths and hSPEPS are shown. For all elements, rf-field strengths values and mixing times indicate the experimentally determined optimum.

In Figure S15 below: SW – spectral width; TD – the number of points in the FID; IN_F – an increment time; AQ – the acquisition time. 2 Dummy Scans were used.

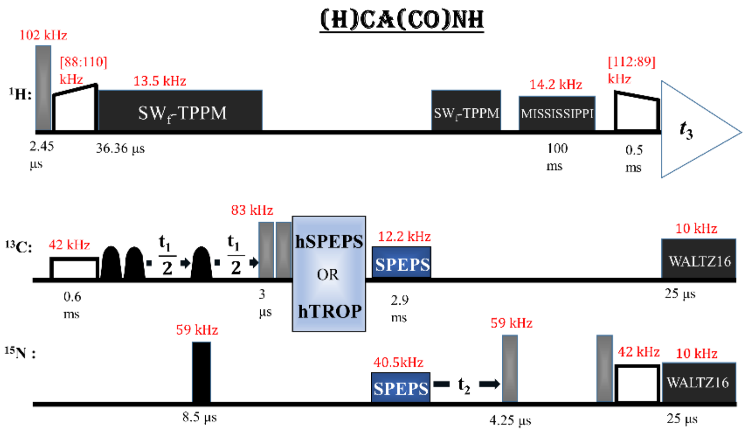

(H)CA(CO)NH	F3	F2	F1
	^{1}H	^{15}N	^{13}C
TD	2048	92	128
SW(ppm)	40.4451	40	40
IN_F(µs)		205.5372	82.8324
AQ(s)	0.0210944	0.0094547	0.0053013
CF	4.7	117	53.7

Number Scans: 8
Recycle delay: 0.3 s
Total Time of the experiment: 11.46 hours

Figure S22 The 3D (H)CA(CO)NH sequence and the experimental parameters (Figure Figure S8B). Red numbers represent rf-field power in kHz. The hard pulse durationss are shown in µs, while total duration of CP and decoupling are in ms. For ^{1}H→^{13}C transfers, CP with [80%:100%] ramp was applied on the proton channel. For ^{15}N→^{1}H transfers, CP with [100%:80%] ramp was applied on the proton channel. For ramped CP, rectanglular brackets indicate the minimal and maximal rf-field of ramped rf-field strengths. The details of ^{1}H→^{13}CA, ^{13}CA→^{15}N and ^{15}N→^{1}H transfers (the shapes, the rf-field strengths and mixing times) are summarized in Tables S3.

Table S4 summarizes the experimental parameters of recoupling elements used for the 3D (H)CO(CA)NH spectrum of S31N M2 (**Figures 2B in the main text**). Additional parameters are shown in Figure S23.

**3D (H)CO(CA)NH**		hTROP	hSPEPS
^{1}H→^{13}CA	ν_{rf}(^{1}H)	linear ramp: [80%:100%] [85:106] (kHz)	linear ramp: [80%:100%] [85:106] (kHz)
	ν_{rf}(^{13}C)	42 kHz	42 kHz
	t_{mix}	1.5 ms	1.5 ms
**^{13}CA→^{13}CO**	ν_{rf}(^{13}C)	**59.7 kHz**	**47.5 kHz**
	t_{mix}	**1.8 ms**	**1.16 ms**
	ν_{rf}(^{15}N)	40.5 kHz	40.5 kHz
	ν_{rf}(^{13}C)	12.2 kHz	12.2 kHz

^{13}CA→^{15}N	t_{mix}	2.3272 ms	2.3272 ms
^{15}N→^{1}H	ν_{rf}(^{1}H)	linear ramp: [100%:80%] [112:89] (kHz)	linear ramp: [100%:80%] [112:89] (kHz)
	ν_{rf}(^{15}N)	42 kHz	42 kHz
	t_{mix}	0.7 ms	0.7 ms

Table S4 The shapes, nominal rf-field strengths and transfer times for recoupling elements as implemented for ^{1}H-^{13}CA, ^{13}CA-^{15}N and ^{15}N-^{1}H transfers at 55 kHz MAS. For ramped CP, rectanglular brackets indicate the minimal and maximal rf-field of ramped rf-field strengths. For hTROP, the hTROP shape files were downloaded from https://optimal-nmr.net/sequences.html and used without any modification. In the table the maximal hTROP rf-field strengths and hSPEPS are shown. For all elements, rf-field strengths values and mixing times indicate the experimentally determined optimum.

In Figure S15 below: SW – spectral width; TD – the number of points in the FID; IN_F – an increment time; AQ – the acquisition time. 2 Dummy Scans were used.

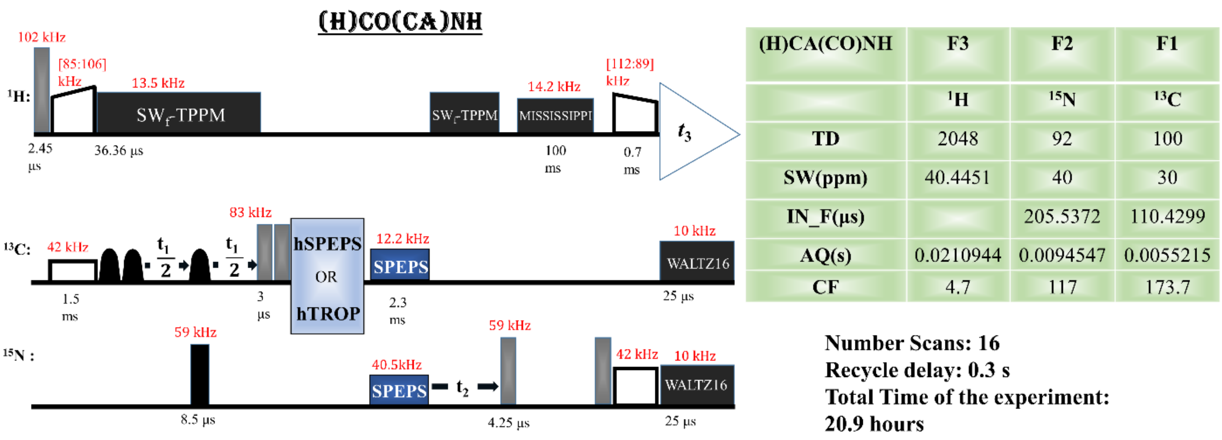

Figure S23 The 3D (H)CO(CA)NH sequence and the experimental parameters (Figure Figure S8B). Red numbers represent rf-field power in kHz. The hard pulse durationss are shown in µs, while total duration of CP and decoupling are in ms. For ^{1}H→^{13}C transfers, CP with [80%:100%] ramp was applied on the proton channel. For ^{15}N→^{1}H transfers, CP with [100%:80%] ramp was applied on the proton channel. For ramped CP, rectanglular brackets indicate the minimal and maximal rf-field of ramped rf-field strengths. The details of ^{1}H→^{13}CA, ^{13}CA→^{15}N and ^{15}N→^{1}H transfers (the shapes, the rf-field strengths and mixing times) are summarized in Tables S4.

1200 MHz, 0.7 mm probe: Experiments acquired at a 1200 MHz on a Bruker Avance NEO spectrometer operating at 28.18 T (1200 MHz ^{1}H frequency) using a 0.7 mm HCN probe. The experiments were performed at 100 kHz MAS, the temperature of the nitrogen cooling gas set to 252 K using 550 liters per hour of flow. For decoupling of the heteronuclear dipolar interactions and water suppression SW$_f$-TPPM,[22] WALTZ-16[24] and MISSISSIPPI[25] were applied. In the Figure below: SW – spectral width; TD – the size of FID; IN_F – an increment time; AQ – the acquisition time. 2 Dummy Scans were used.

Table S5 summarizes the experimental parameters of recoupling elements used for the 3D (H)CA(CO)NH and (HCO)CA(CO)NH spectra of GLPG (**Figure 4 in the main text**). Additional parameters are shown in Figure S24.

**3D (H)CA(CO)NH**<br>**3D (HCO)CA(CO)NH**		J-based	hSPEPS
**Number Scans**		**72**	**24**
**Total time of the experiment**		**10.56 days**	**3.52 days**
^{1}H→^{13}C	ν_{rf}(^{1}H)	linear ramp: [80%:100%]<br>[128:161] (kHz)	linear ramp: [80%:100%]<br>[117:146] (kHz)
	ν_{rf}(^{13}C)	35 kHz	28 kHz
	t_{mix}	2 ms	0.6 ms
**^{13}CA→^{13}CO**	ν_{rf}(^{13}C)	**Train of Soft Pulses**	**102 kHz**
	t_{mix}	**6.67 ms**	**0.96 ms**
^{13}CA→^{15}N	ν_{rf}(^{15}N)	20.6 kHz	22.6 kHz
	ν_{rf}(^{13}C)	79.5 kHz	78 kHz
	t_{mix}	2.88 ms	2.24 ms

$^{15}N \rightarrow ^{1}H$	$\nu_{rf}(^{1}H)$	linear ramp: [100%:80%] [150:120] (kHz)	linear ramp: [100%:80%] [139:111] (kHz)
	$\nu_{rf}(^{15}N)$	23 kHz	23 kHz
	t_{mix}	0.5 ms	0.5 ms

Table S5 The shapes, nominal rf-field strengths and transfer times for recoupling elements as implemented for ^{1}H-^{13}CA, ^{13}CA-^{15}N and ^{15}N-^{1}H transfers at 100 kHz MAS. For ramped CP, rectanglular brackets indicate the minimal and maximal rf-field of ramped rf-field strengths. In the table the maximal hSPEPS are shown. For J-based, a train of soft pulses has been applied as depicted in Figure S15. For all elements, rf-field strengths values and mixing times indicate the experimentally determined optimum.

In Figure S24 below: SW – spectral width; TD – the number of points in the FID; IN_F – an increment time; AQ – the acquisition time. 2 Dummy Scans were used.

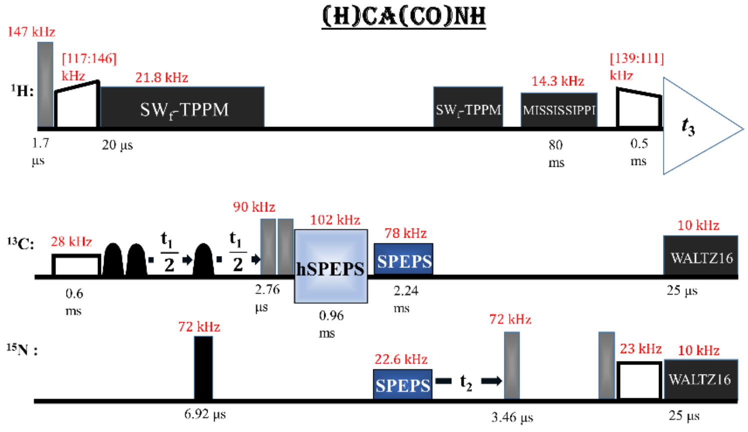

(H)CA(CO)NH	F3	F2	F1
	^{1}H	^{15}N	^{13}C
TD	1386	102	110
SW(ppm)	46.3664	40	35
IN_F(µs)		205.5374	94.6656
AQ(s)	0.0210944	0.0104824	0.0052066
CF	4.8	116	53.7

Number Scans: 24
Recycle delay: 1 s
Total Time of the experiment: 3.52 days

Figure S24 The 3D (H)CA(CO)NH sequence and the experimental parameters (Figure Figure S8B). Red numbers represent rf-field power in kHz. The hard pulse durationss are shown in µs, while total duration of CP and decoupling are in ms. For ^{1}H→^{13}C transfers, CP with [80%:100%] ramp was applied on the proton channel. For ^{15}N→^{1}H transfers, CP with [100%:80%] ramp was applied on the proton channel. For ramped CP, rectanglular brackets indicate the minimal and maximal rf-field of ramped rf-field strengths. The details of ^{1}H→^{13}CA, ^{13}CA→^{15}N and ^{15}N→^{1}H transfers (the shapes, the rf-field strengths and mixing times) are summarized in Tables S5.

Compiling script (Echo/ Anti-Echo mode into STATES/STATES-TPPI mode).

The Echo/Anti-Echo → STATES /STATES-TTPI conversion script[5] was incorporated into a portion of the drift correction script, which was origionally written by Najbauer and Andreas.[26] It handles more complex cases as compared with the default processing, as presented before.[27] The conversion is implemented before the drift correction part. If there is no requirement for drift correction, both 'the start shift' and 'the end shift' values are set to zero.

For 2D and 3D experiments with a single Echo/Anti-Echo mode for indirect (t_1) dimension, the script faithfully converts the experimental data only for a specific phase cycling setup of a pair of 90°-pulses (inversion real part before element). In the case of 3D experiments with Echo/Anti-Echo modes for (t_1) and (t_2) indirect dimensions, the first pair of a 90°-pulses should invert the real part and the second should invert the imaginary. The order of fids, when acquired as planes mode should be 321 (numbers indicate the indexing of the dimensions, F1,F2,F3).

BRUKER PULSE PROGRAMS

The widths of hSPEPS and SPEPS pulses are automatically calculated using the 'cnst31' parameter (the MAS rate in Hz). 'cnst31' should have the same exactly MAS rate value, used in the experiment.

2D (H)CC

```
;2D hCC carbon detect experiment with hSPEPS^{CA-CB} sequence
;with 15N and 13C decoupling
;Avance II+ version
;parameters:
;p1 : 13C 90 pulse @ plw1
;pl1 : 13C power for 90 pulse

;spnam0 : Ramp80.100
;spoffs0 : 0

;p15 : contact time for H->C CP
;pl20 : 13C power for CP
;sp6 : 1H power for H->C CP
```

```
;spnam10 : Ramp80.100
;spoffs10 : 0

;p7 : 15N 90 pulse @ plw7
;pl7 : 15N power for 90 pulse

;sp5 : homo-SPEPS; 1.25*MAS rate
;l20 : Number of hSPEPS mixing blocks

;p2 : 1H 90 pulse @ plw2
;pl2 : 1H power for 90 pulse


;cpdprg2 : 1H decoupling (sltppm_40pTr41 for 1Hprot, waltz16_pl12 for 2Hprot)
;cpdprg3 : 15N decoupling (waltz16_pl17)

;pl12 : 1H decoupling)
;pl18 : 1H decoupling for (cw_pl18)
;pl13: water suppression

; 15N settings
"pcpd3=25"                    ;does not work!
"plw17=plw7*(pow(p7/25,2))"     ; 15N waltz 10kHz decoupling power level
"spoal3=0.5"   ; default value (irrelevant)
"spoff3=0.0"     ; on-resonance


;pcpd4 : 33.33 (sltppm 15kHz)
;pcpd2 : 25u (waltz16 10 kHz) - 33.33 (sltppm 15kHz)


;$COMMENT=basic cp experiment, arbitrary contact and decoupling schemes
;$CLASS=Solids
;$TYPE=cross polarisation
;$SUBTYPE=simple 1D
;$OWNER=Bruker
prosol relations=<solids_cp>

#include <Avancesolids.incl>

;cnst20 : RF field achieved at pl13
;cnst21 : on resonance, usually = 0
"acqt0=1u*cnst11"
"in0=inf1"

"p5 = ((16s/cnst31))"

define delay mix
```

```
"mix = (l20*p5)"

1m
  if "p5   > 2000u"    goto Problem
  if "mix   > 20m"    goto Problem
  if "p15   > 15m"  goto Problem
  if "aq    > 45m"    goto Problem
  goto PassParams
Problem, 1m
  print "Parameters not accepted, ending."
  goto HaltAcqu
PassParams, 1m

1 ze
  mix
2 d1 do:f2 do:f3
#include <p15_prot.incl>
#include <aq_prot.incl>
  (p2  pl2 ph1):f2
  (p15:sp1 ph2):f1 (p15:sp0 ph10):f2

  1u cpds2:f2
if "p7*2 > d0" goto RAWEVOL
   (center (d0) (p7*2 ph0 pl7):f3)
if "p7*2 <= d0" goto DECOFF

RAWEVOL, 1u
  d0

DECOFF, 1u do:f2

; echo-antiecho determination
(p1 pl1 ph21):f1        ; 1st half of 180
(p1 pl1 ph22):f1        ; 2nd half of 180

3
  (p5:sp5 ph5):f1
  lo to 3 times l20

  1u cpds2:f2 cpds3:f3
  go=2 ph31
  1m do:f2  do:f3
  10m mc #0 to 2
  F1EA(calph(ph22, +180),caldel(d0, +in0) & calph(ph2, +180) & calph(ph31, +180))
HaltAcqu, 1m      ;jump address for protection files
exit              ;quit

ph0= 0
```

ph1= 1 3
ph2= 0 0 2 2
ph10= 0

ph5= 0 0 0 0 3 3 3 3
ph31= 1 3 3 1

ph21= 1
ph22= 3

2D (H)CACO

;2D hCC carbon detect experiment with hSPEPS^{CA-CO} sequence
;with 15N and 13C decoupling
;Avance II+ version
;parameters:
;p1 : 13C 90 pulse @ plw1
;pl1 : 13C power for 90 pulse

;spnam0 : Ramp90.100
;spoffs0 : 0

;p15 : contact time for H->C CP
;pl20 : 13C power for CP
;sp6 : 1H power for H->C CP
;spnam10 : Ramp80.100
;spoffs10 : 0

;p7 : 15N 90 pulse @ plw7
;pl7 : 15N power for 90 pulse

;sp5 : homo-SPEPS; 1*MAS rate
;l20 : Number of hSPEPS mixing blocks

;p2 : 1H 90 pulse @ plw2
;pl2 : 1H power for 90 pulse

;cpdprg2 : 1H decoupling (sltppm_40pTr41 for 1Hprot, waltz16_pl12 for 2Hprot)
;cpdprg3 : 15N decoupling (waltz16_pl17)

;pl12 : 1H decoupling)
;pl18 : 1H decoupling for (cw_pl18)
;pl13: water suppression

; 15N settings
"pcpd3=25"   ;does not work!
"plw17=plw7*(pow(p7/25,2))"   ; 15N waltz 10kHz decoupling power level
"spoal3=0.5"   ; default value (irrelevant)
"spoff3=0.0"   ; on-resonance

```
;pcpd4 : 33.33 (sltppm 15kHz)
;pcpd2 : 25u (waltz16 10 kHz) - 33.33 (sltppm 15kHz)


;$COMMENT=basic cp experiment, arbitrary contact and decoupling schemes
;$CLASS=Solids
;$TYPE=cross polarisation
;$SUBTYPE=simple 1D
;$OWNER=Bruker
prosol relations=<solids_cp>

#include <Avancesolids.incl>

;cnst20 : RF field achieved at pl13
;cnst21 : on resonance, usually = 0
;cnst11 : to adjust t=0 for acquisition, if digmod = baseopt
"acqt0=1u*cnst11"
"in0=inf1"
"in30=inf1"

"p5 = ((32s/cnst31))"

define delay mix
"mix = (l20*p5)"

1m
  if "p5   > 2000u"    goto Problem
  if "mix   > 20m"    goto Problem
  if "p15   > 15m"  goto Problem
  if "aq    > 45m"    goto Problem
  goto PassParams
Problem, 1m
  print "Parameters not accepted, ending."
  goto HaltAcqu
PassParams, 1m

1 ze
  mix

2 d1 do:f2 do:f3
#include <p15_prot.incl>
#include <aq_prot.incl>
  1u fq=cnst21(ppm):f1
  (p2  pl2 ph1):f2
  (p15:sp1 ph2):f1 (p15:sp0 ph10):f2
```

```
  1u cpds2:f2
if "p7*2 > d0" goto RAWEVOL
   (center (d0) (p7*2 ph0 pl7):f3)
if "p7*2 <= d0" goto DECOFF

RAWEVOL, 1u
  d0

DECOFF, 1u do:f2

; echo-antiecho determination
(p1 pl1 ph21):f1       ; 1st half of 180
(p1 pl1 ph22):f1       ; 2nd half of 180
 1u fq=cnst19(ppm):f1
3
  (p5:sp5 ph5):f1
  lo to 3 times l20
1u fq=cnst21(ppm):f1
  1u cpds2:f2 cpds3:f3
  go=2 ph31
  1m do:f2  do:f3
  10m mc #0 to 2
  F1EA(calph(ph22, +180),caldel(d0, +in0) & calph(ph2, +180) & calph(ph31, +180))
HaltAcqu, 1m      ;jump address for protection files
exit              ;quit

ph0= 0
ph1= 1 3
ph2=  0 0 2 2
ph10= 0

ph5= 0 0 0 0 1 1 1 1
ph31= 1 3 3 1

ph21= 1
ph22= 3
```

3D (H)CA(CO)NH

```
; 3D (H)CAcoNH with SPEPS element for CO-N transfer and hSPEPS for CAco transfer
; Developed by E. Nimerovsky at the MPI-NAT in the group of L. Andreas
; Based on conventions of the G. Pintacuda Group

;Avance III version
;parameters:
;p1 : 1H 90 pulse duration
;p3 : 13C 90 pulse duration
;p7 : 15N 90 pulse duration
;p30 : water suppression time (30-200 ms)
```

```
;cnst21 : CO offset in ppm (173.7)
;cnst22 : CA offset in ppm (53.7)
;cnst23 : CO/CO offset in ppm (113.7)
;d1 : recycle delay
;d0 : C incremental delay (t1)
;d10 : N incremental delay (t2)
;in10    : 1/2 increment for 15N evolution
;in0   : 1/2 increment for 13CA evolution
;cpdprg1 : tppm (at pl13) or waltz (at pl13)
;cpdprg4 : cwY (at pl12)
;cpdprg5 : cwX (at pl12)
;pcpd1   : pulse length in decoupling sequence (2xtau_r for tppm, 25us for 10kHz waltz)
;pl1    : power level of 1H hard pulse
;pl12   : power level of decoupling (10-15 kHz)
;pl13   : power level for water suppression
;spnam1  : 1H shape for 1H->13CA CP (ramp 10-20%)
;spoal1  : N/A
;spoff1 : [ON/RES]
;sp1    : 1H power level during 1H->13CA CP
;spnam10 : 1H shape for 15N->1H(N) CP (ramp 10-20%)
;spoal10  : N/A
;spoff10 : [ON/RES]
;sp10    : 1H power level during 15N->1H CP
;cpdprg2 : 15N decoupling pattern during acq (waltz-16)
;p17     : contact time 15N->1H(N) CP (300-500 us)
;pcpd2   : pulse length in 15N decoupling sequence (25 us)
;pl7    : power level for 15N hard pulse
;pl16   : power level for 15N decoupling (corr. to 10 kHz)
;pl2    : power level for 15N hard pulse
;pl20   : 15N power level for 15N->1H CP
;spnam2  : 15N shape for 13CA->15N CP (tan-c100-w10pct)
;sp2    : 15N power level for 13CA->15N CP
;spoal2  : N/A
;spoff2 : [ON/RES]
;p15     : contact time 1H->13CA CP (5 ms)
;p16     : contact time 13CA->15N CP (10 ms)
;p18     : Q3 CO pulse duration
;p19     : Q3 CA pulse duration
;p21     : Q5 CO pulse duration
;pcpd3   : pulse length in 13C decoupling sequence (25 us)
;cpdprg3 : 13CO/CA decoupling pattern during 15N evol (waltz-16)
;pl3    : power level of 13C hard pulse [REFERENCE]
;pl17   : power level for 13CO/CA decoupling (10 kHz)
;spnam9  : 13C shape for 13CA->15N CP (rectangle)
;sp9     : 13C power for 13CA->15N CP
;spoal9  : N/A
;spoff9 : [ON/RES CO]
;spnam18 : 13CO selective pulse shape (Q3)
```

```
;spoal18  : N/A
;spoff18 : [ON/RES CO]
;spnam19 : 13CA selective pulse shape (Q3)
;spoal19  : N/A
;spoff19 : [ON/RES CA]

;spnam21 : 13CO selective pulse shape (Q5)
;spoal21  : N/A
;spoff21 : [ON/RES CO]

;spnam29 : 13C shape for 1H->13CO CP (rectangle)
;sp29   : 13C power level for 1H->13CO CP
;spoal29  : N/A
;spoff29 : [ON/RES CO]
;zgoptns : -DFILTER or blank
;td1 : number of C increments
;td2 : number of N increments
;pl9: 13C hard pulse
;l21:  Number SPEPS elements
;sp5: ~0.25*MAS C for SPEPS
;sp6: ~0.75*MAS N for SPEPS

;sp8 : homo-SPEPS; ~MAS rate
;l20 : Number of hSPEPS mixing blocks

;$COMMENT=Inverse Cp with INEPT CBCA mixing
;$CLASS=Solids
;$DIM=3D
;$TYPE=H detect
;$SUBTYPE=Heteronuclear
;$OWNER=CRMN

#include <Avancesolids.incl>

; Start evolutions from exactly 0
"d10=0.0"
"d0=0.0"

; 1H settings
"spoal1=0.5"   ; default value (irrelevant)
"spoff1=0.0"    ; on-resonance
"spoal10=0.5"  ; default value (irrelevant)
"spoff10=0.0"   ; on-resonance

; 15N settings
"pcpd2=25"                      ;does not work!
"plw2=plw7"
"plw17=plw2*(pow(p7/25,2))"     ; 15N waltz 10kHz decoupling power level
```

```
"spoal2=0.5"   ; default value (irrelevant)
"spoff2=0.0"     ; on-resonance

; 13C settings
"plw16=plw3*(pow(p3/25,2))"     ; 13C waltz 10kHz decoupling power level

"cnst22 = (sfo3-bf3)*1000000/bf3"  ; CA frequency offset (ppm)
"cnst21 = cnst22+(173.7-53.7)"     ; CO frequency offset (ppm)
"cnst23 = cnst22+(113.7-53.7)"    ; the offset half-way CO and CA (ppm)

"p18=3.412/(95.0*bf3/1000000)"    ; 95 ppm bandwidth (safe)
"spw18=plw3*pow((0.5/(p18*0.1515))/(0.25/p3),2)" ;  Q3 power level
"spoal18=0.5"                    ; default value (irrelevant)
"spoff18=bf3*((cnst21-cnst22)/1000000)"

"p19=3.412/(105.0*bf3/1000000)"      ; 95 ppm bandwidth (safe)
"spw19=plw3*pow((0.5/(p19*0.1515))/(0.25/p3),2)" ;  Q3 power level
"spoal19=0.5"                          ; default value (irrelevant)
"spoff19=0.0"                    ; CA frequency

"p21=6.18/(105.0*bf3/1000000)"    ; CA: 105 ppm bandwidth (safe)
"spw21=plw3*pow((0.25/(p21*0.05451))/(0.25/p3),2)" ;  Q5 power level
"spoal21=0.5"                    ; default value (irrelevant)
"spoff21=bf3*((cnst21-cnst22)/1000000)" ; CO frequency

"spoal9=0.0"                     ; needed for offset on C
"spoff9=0.0"                     ; on-resonance

"spoal29=1.0"                    ; needed for offset on C
"spoff29=0.0"                    ; on-resonance CA


"in0=inf1/2"
"in10=inf2/2"


;"acqt0=0"              ; baseopt correction

"p10 = (16*(2s/cnst31))"

define delay mix
"mix = (l21*p10)"

"p5 = ((32s/cnst31))"

define delay mix2
"mix2 = (l20*p5)"
```

```
1m
  if "p15   > 15m"     goto Problem
  if "aq    > 56m"     goto Problem
  if "p17    > 7m"     goto Problem
  if "mix    > 15m"    goto Problem
  if "mix2   > 10m"    goto Problem
  goto PassParams
Problem, 1m
  print " cnst31 is too low; aq, p15 or p17 are too long."
  goto HaltAcqu
PassParams, 1m

1 ze
  mix
  mix2
2 d1 do:f2
#include <p15_prot.incl>
#include <aq_prot.incl>

  ;1u fq=0:f3
  1u fq=cnst22(bf ppm):f3
  (p1 pl1 ph3):f1

  (p15:sp29 ph15):f3 (p15:sp1 ph16):f1
# ifdef FILTER
;   Filter for removing h->CO artifacts
  (p3 pl3 ph1):f3
  (p21:sp21 ph0):f3    ; CO selective Pi/2
  10m
  (p3 pl3 ph19):f3
;   end Filter
# endif
  1u cpds1:f1
  (p18:sp18 ph23):f3  ;CO selective Pi
  1u
  (p19:sp19 ph2):f3   ;CA selective Pi
  1u
  d0
  (center (p7*2 ph0 pl7):f2 (p18:sp18 ph2):f3)  ;CO selective Pi
  d0
  1u do:f1

; echo-antiecho determination
(p3 pl3 ph4):f3 ; 1st half of 180
(p3 pl3 ph25):f3 ; 2nd half of 180
1u fq=cnst23(bf ppm):f3
; COca homonuclear mixing
3
```

```
 (p5:sp8 ph8):f3
 lo to 3 times l20
0.5u fq=cnst21(bf ppm):f3 ;move offset from CA to CO
4
 (p10:sp5 ph10):f3 (p10:sp6 ph12):f2
 lo to 4 times l21
 1u fq=cnst23(bf ppm):f3
 1u cpds1:f1
 d10
 (p3*2 pl3 ph0):f3
 d10
 1u do:f1
 (p7 pl7 ph5):f2
;water suppression
 (p30*0.25 pl13 ph0):f1
 (p30*0.25 pl13 ph1):f1
 (p30*0.25 pl13 ph0):f1
 (p30*0.25 pl13 ph1):f1
;water suppression
 (p7 pl7 ph6):f2

 (p17 pl20 ph7):f2 (p17:sp10 ph17):f1

 1u cpds2:f2
 go=2 ph31
 1m do:f2

 10m mc #0 to 2

F1EA(calph(ph25, +180) ,caldel(d0, +in0) & calph(ph15, +180) & calph(ph31, +180))  ;13C
F2PH(calph(ph5, +90), caldel(d10, +in10)) ;15N

HaltAcqu, 1m      ;jump address for protection files
exit              ;quit

ph0  = 0
ph1  = 1
ph3  = 1
ph15 = 2
ph16 = 0 0 2 2
ph23= 0 1 0 1 1 0 1 0
ph5 =  1
ph6 =  3
ph2 = 0
ph4= 1
ph25= 3
ph10= 0 3
ph12= 0 1
```

```
ph21= 0
ph22= 2
ph8= 0 0 0 0 1 1 1 1
ph7  = 2 2 2 2 0 0 0 0
ph17 = 0
ph19=3

ph31 = 1 3 3 1 3 1 1 3
```

3D (H)CO(CA)NH

```
;3D (H)COcaNH with SPEPS element for CA-N transfer and hSPEPS for COca transfer
; Developed by E. Nimerovsky at the MPI-NAT in the group of L. Andreas
; Based on conventions of the G. Pintacuda Group


; Recent notes:
; this version does not require 13C axis inversion

;Avance III version
;parameters:
;p1 : 1H 90 pulse duration
;p3 : 13C 90 pulse duration
;p7 : 15N 90 pulse duration
;p30 : water suppression time (30-200 ms)
;cnst21 : CO offset in ppm (173.7)
;cnst22 : CA offset in ppm (53.7)
;cnst23 : CO/CO offset in ppm (113.7)
;d1 : recycle delay
;d0 : C incremental delay (t1)
;d10 : N incremental delay (t2)
;in10    : 1/2 increment for 15N evolution
;in0   : 1/2 increment for 13CA evolution
;cpdprg1 : tppm (at pl13) or waltz (at pl13)
;cpdprg4 : cwY (at pl12)
;cpdprg5 : cwX (at pl12)
;pcpd1   : pulse length in decoupling sequence (2xtau_r for tppm, 25us for 10kHz waltz)
;pl1    : power level of 1H hard pulse
;pl12   : power level of decoupling (10-15 kHz)
;pl13   : power level for water suppression
;spnam1  : 1H shape for 1H->13CA CP (ramp 10-20%)
;spoal1  : N/A
;spoff1 : [ON/RES]
;sp1    : 1H power level during 1H->13CO CP
;spnam10 : 1H shape for 15N->1H(N) CP (ramp 10-20%)
;spoal10  : N/A
;spoff10 : [ON/RES]
;sp10    : 1H power level during 15N->1H CP
```

```
;cpdprg2 : 15N decoupling pattern during acq (waltz-16)
;p17     : contact time 15N->1H(N) CP (300-500 us)
;pcpd2   : pulse length in 15N decoupling sequence (25 us)
;pl7    : power level for 15N hard pulse
;pl16   : power level for 15N decoupling (corr. to 10 kHz)
;pl2    : power level for 15N hard pulse
;pl20   : 15N power level for 15N->1H CP
;spnam2  : 15N shape for 13CA->15N CP (tan-c100-w10pct)
;sp2    : 15N power level for 13CA->15N CP
;spoal2  : N/A
;spoff2 : [ON/RES]
;p15     : contact time 1H->13CO CP (5 ms)
;p16     : contact time 13CA->15N CP (10 ms)
;p18     : Q3 CO pulse duration
;p19     : Q3 CA pulse duration
;pcpd3   : pulse length in 13C decoupling sequence (25 us)
;cpdprg3 : 13CO/CA decoupling pattern during 15N evol (waltz-16)
;pl3    : power level of 13C hard pulse [REFERENCE]
;pl17   : power level for 13CO/CA decoupling (10 kHz)
;spnam9  : 13C shape for 13CA->15N CP (rectangle)
;sp9     : 13C power for 13CA->15N CP
;spoal9  : N/A
;spoff9 : [ON/RES CO]
;spnam18 : 13CO selective pulse shape (Q3)
;spoal18  : N/A
;spoff18 : [ON/RES CO]
;spnam19 : 13CA selective pulse shape (Q3)
;spoal19  : N/A
;spoff19 : [ON/RES CA]
;spnam29 : 13C shape for 1H->13CO CP (rectangle)
;sp29   : 13C power level for 1H->13CO CP
;spoal29  : N/A
;spoff29 : [ON/RES CO]
;zgoptns : -Dfslg, -Dlacq, or blank
;td1 : number of C increments
;td2 : number of N increments

;l21:  Number SPEPS elements
;sp5: ~0.25*MAS C for SPEPS
;sp6: ~0.75*MAS N for SPEPS

;sp8 : homo-SPEPS; ~MAS rate
;l20 : Number of hSPEPS mixing blocks

;$COMMENT=Inverse Cp with INEPT CBCA mixing
;$CLASS=Solids
;$DIM=3D
;$TYPE=H detect
```

```
;$SUBTYPE=Heteronuclear
;$OWNER=CRMN

#include <Avancesolids.incl>

; Start evolutions from exactly 0
"d10=0.0"
"d0=0.0"

; 1H settings
"spoal1=0.5"   ; default value (irrelevant)
"spoff1=0.0"    ; on-resonance
"spoal10=0.5"  ; default value (irrelevant)
"spoff10=0.0"   ; on-resonance

; 15N settings
"pcpd2=25"                    ;does not work!
"plw2=plw7"
"plw16=plw2*(pow(p7/25,2))"     ; 15N waltz 10kHz decoupling power level
"spoal2=0.5"   ; default value (irrelevant)
"spoff2=0.0"     ; on-resonance

; 13C settings
"plw17=plw3*(pow(p3/25,2))"     ; 13 waltz 10kHz decoupling power level

"cnst21 = (sfo3-bf3)*1000000/bf3"  ; CO frequency offset (ppm)
"cnst22 = cnst21-(173.7-53.7)"     ; CA frequency offset (ppm)
"cnst23 = cnst21-(173.7-113.7)"    ; the offset half-way CO and CA (ppm)

"p18=3.412/(95.0*bf3/1000000)"    ; 95 ppm bandwidth (safe)
"spw18=plw3*pow((0.5/(p18*0.1515))/(0.25/p3),2)" ;  Q3 power level
"spoal18=0.5"                    ; default value (irrelevant)
"spoff18=0.0"

"p19=3.412/(105.0*bf3/1000000)"      ; 105 ppm bandwidth (safe)
"spw19=plw3*pow((0.5/(p19*0.1515))/(0.25/p3),2)" ;  Q3 power level
"spoal19=0.5"                         ; default value (irrelevant)
"spoff19=bf3*((cnst22-cnst21)/1000000)" ; CA frequency



"spoal8=0.5"                     ; default value (irrelevant)
"spoff8=0.0"                      ; on-resonance

"spoal29=1.0"                     ; default value (irrelevant)
"spoff29=0.0"                      ; on-resonance

"in0=inf1/2"
```

```
"in10=inf2/2"


;"acqt0=0"              ; baseopt correction

"p10 = (16*(2s/cnst31))"

define delay mix
"mix = (l21*p10)"

"p5 = ((32s/cnst31))"

define delay mix2
"mix2 = (l20*p5)"

define delay del26
"del26 = p24 + p25 + 1u"

1m
  if "p15   > 15m"     goto Problem
  if "aq    > 56m"     goto Problem
  if "p17    > 7m"     goto Problem
  if "mix    > 15m"    goto Problem
  if "mix2   > 10m"    goto Problem
  goto PassParams
Problem, 1m
  print " cnst31 is too low; aq, p15 or p17 are too long."
  goto HaltAcqu
PassParams, 1m

1 ze
  mix
  mix2
2 d1 do:f2
#include <p15_prot.incl>
#include <aq_prot.incl>

  ;1u fq=0:f3
  1u fq=cnst21(bf ppm):f3   ;go back to the CO frequency
  (p1 pl1 ph3):f1

  (p15:sp29 ph15):f3 (p15:sp1 ph16):f1

  1u cpds1:f1
  (p19:sp19 ph23):f3  ;CA selective Pi
  1u
  (p18:sp18 ph2):f3   ;CO selective Pi
  1u
```

```
  d0
  (center (p7*2 ph0 pl7):f2 (p19:sp19 ph2):f3)  ;CA selective Pi
  d0
  1u do:f1
; echo-antiecho determination
(p3 pl3 ph4):f3        ; 1st half of 180
(p3 pl3 ph25):f3       ; 2nd half of 180
0.5u fq=cnst23(bf ppm):f3
; COca homonuclear mixing
3
  (p5:sp8 ph8):f3
  lo to 3 times l20
    0.5u fq=cnst22(bf ppm):f3 ;move offset from CO to CA
4
  (p10:sp5 ph10):f3 (p10:sp6 ph12):f2
  lo to 4 times l21
  1u fq=cnst23(bf ppm):f3
  1u cpds1:f1
  d10
  (p3*2 pl3 ph0):f3
  d10
  1u do:f1
  (p7 pl7 ph5):f2
 ;water suppression
  (p30*0.25 pl13 ph0):f1
  (p30*0.25 pl13 ph1):f1
  (p30*0.25 pl13 ph0):f1
  (p30*0.25 pl13 ph1):f1
 ;water suppression
  (p7 pl7 ph6):f2

  (p17 pl20 ph7):f2 (p17:sp10 ph17):f1

  1u cpds2:f2
  go=2 ph31
  1m do:f2

  10m mc #0 to 2

 F1EA(calph(ph25, +180),caldel(d0, +in0) & calph(ph15, +180) & calph(ph31, +180))  ;13C
 F2PH(calph(ph5, +90), caldel(d10, +in10)) ;15N

HaltAcqu, 1m      ;jump address for protection files
exit              ;quit

ph0  = 0
ph1  = 1
ph3  = 1
```

ph15 = 2
ph16 = 0 0 2 2
ph5 = 1
ph6 = 3
ph2 = 0
ph4= 1
ph25= 3
ph10= 0 3
ph12= 0 1
ph21= 0
ph22= 2
ph8= 0 0 0 0 1 1 1 1
ph7= 2 2 2 2 0 0 0 0
ph17 = 0

ph23= 0 1 0 1 1 0 1 0
ph24= 0
ph26= 1 3
ph27= 1
ph31 = 1 3 3 1 3 1 1 3

3D m(H)CO(CA)NH

; 3D modified (H)COcaNH with SPEPS element for CA-N transfer and hSPEPS for COca transfer
; Developed by E. Nimerovsky at the MPI-NAT in the group of L. Andreas
; Based on conventions of the G. Pintacuda Group

; CONTAINS ADDITIONAL BLOCK OF SOFT PULSES TO ELIMINATE CO-N TRANSFERS DURING
; ^{13}CA-^{15}N SPEPS TRANSFER STEP.

; Recent notes:
; this version does not require 13C axis inversion

;Avance III version
;parameters:
;p1 : 1H 90 pulse duration
;p3 : 13C 90 pulse duration
;p7 : 15N 90 pulse duration
;p30 : water suppression time (30-200 ms)
;cnst21 : CO offset in ppm (173.7)
;cnst22 : CA offset in ppm (53.7)
;cnst23 : CO/CO offset in ppm (113.7)
;d1 : recycle delay
;d0 : C incremental delay (t1)
;d10 : N incremental delay (t2)
;in10   : 1/2 increment for 15N evolution
;in0   : 1/2 increment for 13CA evolution
;cpdprg1 : tppm (at pl13) or waltz (at pl13)
;pcpd1   : pulse length in decoupling sequence (2xtau_r for tppm, 25us for 10kHz waltz)

```
;pl1    : power level of 1H hard pulse
;pl12   : power level of decoupling (10-15 kHz)
;pl13   : power level for water suppression
;spnam1  : 1H shape for 1H->13CA CP (ramp 10-20%)
;spoal1  : N/A
;spoff1 : [ON/RES]
;sp1    : 1H power level during 1H->13CA CP
;spnam10 : 1H shape for 15N->1H(N) CP (ramp 10-20%)
;spoal10  : N/A
;spoff10 : [ON/RES]
;sp10    : 1H power level during 15N->1H CP
;cpdprg2 : 15N decoupling pattern during acq (waltz-16)
;p17     : contact time 15N->1H(N) CP (300-500 us)
;pcpd2   : pulse length in 15N decoupling sequence (25 us)
;pl7    : power level for 15N hard pulse
;pl16   : power level for 15N decoupling (corr. to 10 kHz)
;pl2    : power level for 15N hard pulse
;pl20   : 15N power level for 15N->1H CP
;spnam2  : 15N shape for 13CA->15N CP (tan-c100-w10pct)
;sp2    : 15N power level for 13CA->15N CP
;spoal2  : N/A
;spoff2 : [ON/RES]
;p15     : contact time 1H->13CA CP (5 ms)
;p16     : contact time 13CA->15N CP (10 ms)
;p18     : Q3 CO pulse duration
;p19     : Q3 CA pulse duration
;pcpd3   : pulse length in 13C decoupling sequence (25 us)
;cpdprg3 : 13CO/CA decoupling pattern during 15N evol (waltz-16)
;pl3    : power level of 13C hard pulse [REFERENCE]
;pl17   : power level for 13CO/CA decoupling (10 kHz)
;spnam9  : 13C shape for 13CA->15N CP (rectangle)
;sp9     : 13C power for 13CA->15N CP
;spoal9  : N/A
;spoff9 : [ON/RES CO]
;spnam18 : 13CO selective pulse shape (Q3)
;spoal18  : N/A
;spoff18 : [ON/RES CO]
;spnam19 : 13CA selective pulse shape (Q3)
;spoal19  : N/A
;spoff19 : [ON/RES CA]
;spnam29 : 13C shape for 1H->13CO CP (rectangle)
;sp29   : 13C power level for 1H->13CO CP
;spoal29  : N/A
;spoff29 : [ON/RES CO]
;zgoptns : -Dfslg, -Dlacq, or blank
;td1 : number of C increments
;td2 : number of N increments
```

```
;l21:  Number SPEPS elements
;sp5: ~0.25*MAS C for SPEPS
;sp6: ~0.75*MAS N for SPEPS

;sp8 : homo-SPEPS; ~MAS rate
;l20 : Number of hSPEPS mixing blocks

;$CLASS=Solids
;$DIM=3D
;$TYPE=H detect
;$SUBTYPE=Heteronuclear
;$OWNER=CRMN

#include <Avancesolids.incl>

; Start evolutions from exactly 0
"d10=0.0"
"d0=0.0"

; 1H settings
"spoal1=0.5"   ; default value (irrelevant)
"spoff1=0.0"    ; on-resonance
"spoal10=0.5"  ; default value (irrelevant)
"spoff10=0.0"   ; on-resonance

; 15N settings
"pcpd2=25"                      ;does not work!
"plw2=plw7"
"plw16=plw2*(pow(p7/25,2))"     ; 15N waltz 10kHz decoupling power level
"spoal2=0.5"   ; default value (irrelevant)
"spoff2=0.0"     ; on-resonance

; 13C settings
"plw17=plw3*(pow(p3/25,2))"     ; 13 waltz 10kHz decoupling power level

"cnst21 = (sfo3-bf3)*1000000/bf3"  ; CO frequency offset (ppm)
"cnst22 = cnst21-(173.7-53.7)"     ; CA frequency offset (ppm)
"cnst23 = cnst21-(173.7-113.7)"    ; the offset half-way CO and CA (ppm)

"p18=3.412/(95.0*bf3/1000000)"    ; 95 ppm bandwidth (safe)
"spw18=plw3*pow((0.5/(p18*0.1515))/(0.25/p3),2)" ;  Q3 power level
"spoal18=0.5"                    ; default value (irrelevant)
"spoff18=0.0"

"p19=3.412/(105.0*bf3/1000000)"      ; 105 ppm bandwidth (safe)
"spw19=plw3*pow((0.5/(p19*0.1515))/(0.25/p3),2)" ;  Q3 power level
"spoal19=0.5"                         ; default value (irrelevant)
"spoff19=bf3*((cnst22-cnst21)/1000000)" ; CA frequency
```

```
"p20=6.18/(105.0*bf3/1000000)"    ; CA: 105 ppm bandwidth (safe)
"spw20=plw3*pow((0.25/(p20*0.05451))/(0.25/p3),2)" ;  Q5 power level
"spoal20=0.5"                    ; default value (irrelevant)
"spoff20=0"                      ; CA on resonance

"p21=6.18/(105.0*bf3/1000000)"    ; CA: 105 ppm bandwidth (safe)
"spw21=plw3*pow((0.25/(p21*0.05451))/(0.25/p3),2)" ;  Q5 power level
"spoal21=0.5"                    ; default value (irrelevant)
"spoff21=0"                      ; CA on resonance

"p22=3.412/(105.0*bf3/1000000)"      ; 105 ppm bandwidth (safe)
"spw22=plw3*pow((0.5/(p22*0.1515))/(0.25/p3),2)" ;  Q3 power level
"spoal22=0.5"                          ; default value (irrelevant)
"spoff22=0.0" ;                       CA frequency

"p24=3.412/(95.0*bf3/1000000)"    ; 95 ppm bandwidth (safe)
"spw24=plw3*pow((0.5/(p24*0.1515))/(0.25/p3),2)" ;  Q3 power level
"spoal24=1.0"                    ; default value (irrelevant)
"spoff24=bf3*((cnst21-cnst22)/1000000)"


"spoal8=0.5"                     ; default value (irrelevant)
"spoff8=0.0"                      ; on-resonance

"spoal29=1.0"                     ; default value (irrelevant)
"spoff29=0.0"                      ; on-resonance

"in0=inf1/2"
"in10=inf2/2"


;"acqt0=0"             ; baseopt correction

"p10 = (16*(2s/cnst31))"

define delay mix
"mix = (l21*p10)"

"p5 = ((32s/cnst31))"

define delay mix2
"mix2 = (l20*p5)"

define delay del26
"del26 = p24"

1m
```

```
  if "p15   > 15m"     goto Problem
  if "aq    > 56m"     goto Problem
  if "p17    > 7m"     goto Problem
  if "mix    > 15m"    goto Problem
  if "mix2   > 10m"    goto Problem
  goto PassParams
Problem, 1m
  print " cnst31 is too low; aq, p15 or p17 are too long."
  goto HaltAcqu
PassParams, 1m

1 ze
  mix
  mix2
2 d1 do:f2
#include <p15_prot.incl>
#include <aq_prot.incl>

  ;1u fq=0:f3
  1u fq=cnst21(bf ppm):f3   ;go back to the CO frequency
  (p1 pl1 ph3):f1

  (p15:sp29 ph15):f3 (p15:sp1 ph16):f1

  1u cpds1:f1
  (p19:sp19 ph23):f3  ;CA selective Pi
  1u
  (p18:sp18 ph2):f3   ;CO selective Pi
  1u
  d0
  (center (p7*2 ph0 pl7):f2 (p19:sp19 ph2):f3)  ;CA selective Pi
  d0
  (p18:sp18 ph27):f3   ;CO selective Pi
  ;(p3*2 pl3 ph27):f3
  1u do:f1
; echo-antiecho determination
(p3 pl3 ph4):f3        ; 1st half of 180
(p3 pl3 ph25):f3        ; 2nd half of 180
0.5u fq=cnst23(bf ppm):f3
; COca homonuclear mixing
3
  (p5:sp8 ph8):f3
  lo to 3 times l20

    1u cpds1:f1
    1u fq=cnst22(bf ppm):f3 ;move offset from middle to CA
    (p24:sp24 ph24):f3    ; 180 CO selective spoal = 0.5
;  del26
```

```
   1u
   (p22:sp22 ph2):f3    ; CA selective Pi
 1u
   (p24:sp24 ph28):f3    ; 180 CO selective spoal = 0.5
   1u
   1u do:f1
4
  (p10:sp5 ph10):f3 (p10:sp6 ph12):f2
  lo to 4 times l21
  1u fq=cnst23(bf ppm):f3
  1u cpds1:f1 cpds3:f3
  d10
  ;(p3*2 pl3 ph0):f3
  d10
  1u do:f1 do:f3
  (p7 pl7 ph5):f2
 ;water suppression
  (p30*0.25 pl13 ph0):f1
  (p30*0.25 pl13 ph1):f1
  (p30*0.25 pl13 ph0):f1
  (p30*0.25 pl13 ph1):f1
 ;water suppression
  (p7 pl7 ph6):f2

  (p17 pl20 ph7):f2 (p17:sp10 ph17):f1

  1u cpds2:f2
  go=2 ph31
  1m do:f2

  10m mc #0 to 2

 F1EA(calph(ph25, +180),caldel(d0, +in0) & calph(ph15, +180) & calph(ph31, +180))  ;13C
 F2PH(calph(ph5, +90), caldel(d10, +in10)) ;15N

HaltAcqu, 1m      ;jump address for protection files
exit              ;quit

ph0  = 0
ph1  = 1
ph3  = 1
ph15 = 2
ph16 = 0 0 2 2
ph5 =  1
ph6 =  3
ph2 = 0
ph4= 1
ph25= 3
```

ph10= 0 3
ph12= 0 1
ph21= 0
ph22= 2
ph8= 0 0 0 0 1 1 1 1
ph7= 2 2 2 2 0 0 0 0
ph17 = 0

ph23= 0 1 0 1 1 0 1 0
ph24= 0 0 1 1
ph28= 0 0 0 0 1 1 1 1
ph26= 1 3
ph27= 1
ph31 = 1 3 3 1 3 1 1 3

3D (H)CB(CA)NH

;3D (H)CB(CA)NH with SPEPS element for CA-N transfer and hSPEPS for CBca transfer
; Developed by E. Nimerovsky at the MPI-NAT in the group of L. Andreas
; Based on conventions of the G. Pintacuda Group

; Recent notes:
; this version does not require 13C axis inversion

;Avance III version
;parameters:
;p1 : 1H 90 pulse duration
;p3 : 13C 90 pulse duration
;p7 : 15N 90 pulse duration
;p30 : water suppression time (30-200 ms)
;cnst21 : CO offset in ppm (173.7)
;cnst22 : CB offset in ppm (39.7)
;cnst23 : CA/CO offset in ppm (113.7)
;cnst24 : CA offset in ppm (53.7)

;d1 : recycle delay
;d0 : C incremental delay (t1)
;d10 : N incremental delay (t2)
;in10 : 1/2 increment for 15N evolution
;in0 : 1/2 increment for 13CA evolution
;cpdprg1 : tppm (at pl13) or waltz (at pl13)
;pcpd1 : pulse length in decoupling sequence (2xtau_r for tppm, 25us for 10kHz waltz)
;pl1 : power level of 1H hard pulse
;pl12 : power level of decoupling (10-15 kHz)
;pl13 : power level for water suppression

```
;spnam1  : 1H shape for 1H->13CA CP (ramp 10-20%)
;spoal1  : N/A
;spoff1 : [ON/RES]
;sp1    : 1H power level during 1H->13CA CP
;spnam10 : 1H shape for 15N->1H(N) CP (ramp 10-20%)
;spoal10  : N/A
;spoff10 : [ON/RES]
;sp10    : 1H power level during 15N->1H CP
;cpdprg2 : 15N decoupling pattern during acq (waltz-16)
;p17     : contact time 15N->1H(N) CP (300-500 us)
;pcpd2   : pulse length in 15N decoupling sequence (25 us)
;pl7    : power level for 15N hard pulse
;pl16   : power level for 15N decoupling (corr. to 10 kHz)
;pl2    : power level for 15N hard pulse
;pl20   : 15N power level for 15N->1H CP
;spnam2  : 15N shape for 13CA->15N CP (tan-c100-w10pct)
;sp2    : 15N power level for 13CA->15N CP
;spoal2  : N/A
;spoff2 : [ON/RES]
;p15     : contact time 1H->13CA CP (5 ms)
;p16     : contact time 13CA->15N CP (10 ms)
;p18     : Q3 CO pulse duration
;p19     : Q3 CA pulse duration
;pcpd3   : pulse length in 13C decoupling sequence (25 us)
;cpdprg3 : 13CO/CA decoupling pattern during 15N evol (waltz-16)
;pl3    : power level of 13C hard pulse [REFERENCE]
;pl17   : power level for 13CO/CA decoupling (10 kHz)
;spnam9  : 13C shape for 13CA->15N CP (rectangle)
;sp9     : 13C power for 13CA->15N CP
;spoal9  : N/A
;spoff9 : [ON/RES CO]
;spnam18 : 13CO selective pulse shape (Q3)
;spoal18  : N/A
;spoff18 : [ON/RES CO]
;spnam19 : 13CA selective pulse shape (Q3)
;spoal19  : N/A
;spoff19 : [ON/RES CA]
;spnam29 : 13C shape for 1H->13CA CP (rectangle)
;sp29   : 13C power level for 1H->13CA CP
;spoal29  : N/A
;spoff29 : [ON/RES CA]
;zgoptns : -Dfslg, -Dlacq, or blank
```

```
;td1 : number of C increments
;td2 : number of N increments

;l21:  Number SPEPS elements
;sp5: ~0.25*MAS C for SPEPS
;sp6: ~0.75*MAS N for SPEPS

;sp8 : homo-SPEPS; ~1.25*MAS rate
;l20 : Number of hSPEPS mixing blocks

;$CLASS=Solids
;$DIM=3D
;$TYPE=H detect
;$SUBTYPE=Heteronuclear
;$OWNER=CRMN

#include <Avancesolids.incl>

; Start evolutions from exactly 0
"d10=0.0"
"d0=0.0"

; 1H settings
"spoal1=0.5"   ; default value (irrelevant)
"spoff1=0.0"    ; on-resonance
"spoal10=0.5"  ; default value (irrelevant)
"spoff10=0.0"   ; on-resonance

; 15N settings
"pcpd2=25"                    ;does not work!
"plw2=plw7"
"plw16=plw2*(pow(p7/25,2))"     ; 15N waltz 10kHz decoupling power level
"spoal2=0.5"   ; default value (irrelevant)
"spoff2=0.0"     ; on-resonance

; 13C settings
"plw17=plw3*(pow(p3/25,2))"     ; 13C waltz 10kHz decoupling power level

"cnst22 = (sfo3-bf3)*1000000/bf3"  ; CB frequency offset (ppm)
"cnst21 = cnst22+(173.7-39.7)"     ; CO frequency offset (ppm)
"cnst23 = cnst22+(113.7-39.7)"    ; the offset half-way CO and CA (ppm)
"cnst24 = cnst22+(53.7-39.7)"     ; CA frequency offset (ppm)
```

```
"p18=3.412/(95.0*bf3/1000000)"    ; 95 ppm bandwidth (safe)
"spw18=plw3*pow((0.5/(p18*0.1515))/(0.25/p3),2)" ;  Q3 power level
"spoal18=0.5"                     ; default value (irrelevant)
"spoff18=bf3*((cnst21-cnst22)/1000000)"

"p19=3.412/(95.0*bf3/1000000)"      ; 95 ppm bandwidth (safe)
"spw19=plw3*pow((0.5/(p19*0.1515))/(0.25/p3),2)" ;  Q3 power level
"spoal19=0.5"                          ; default value (irrelevant)
"spoff19=0.0"                     ; CA frequency

"spoal9=0.0"                      ; needed for offset on C
"spoff9=0.0"                      ; on-resonance

"spoal29=1.0"                     ; needed for offset on C
"spoff29=0.0"                     ; on-resonance CA

"in0=inf1/2"
"in10=inf2/2"


;"acqt0=0"              ; baseopt correction

"p10 = (16*(2s/cnst31))"

define delay mix
"mix = (l21*p10)"

"p10 = (16*(2s/cnst31))"

"p5 = ((16s/cnst31))"

define delay mix2
"mix2 = (l20*p5)"

;aqseq 321

1 ze
  mix
  mix2
2 d1 do:f2
#include <p15_prot.incl>
```

```
#include <aq_prot.incl>

  ;1u fq=0:f3
  1u fq=cnst22(bf ppm):f3
  (p1 pl1 ph3):f1

  (p15:sp29 ph15):f3 (p15:sp1 ph16):f1

  1u cpds1:f1
  (p18:sp18 ph2):f3  ;CO selective Pi
  1u
  (p19:sp19 ph2):f3   ;CA selective Pi
  1u
  d0
  (center (p7*2 ph0 pl7):f2 (p18:sp18 ph2):f3)  ;CO selective Pi
  d0
  1u do:f1

; echo-antiecho determination
(p3 pl3 ph4):f3        ; 1st half of 180
(p3 pl3 ph25):f3        ; 2nd half of 180

3
  (p5:sp8 ph8):f3
  lo to 3 times l20
0.5u fq=cnst24(bf ppm):f3

4 (p10:sp5 ph10):f3 (p10:sp6 ph12):f2
  lo to 4 times l21
  1u fq=cnst23(bf ppm):f3
  1u cpds1:f1
  d10
  (p3*2 pl3 ph0):f3
  d10
  1u do:f1
  (p7 pl7 ph5):f2
 ;water suppression
  (p30*0.25 pl13 ph0):f1
  (p30*0.25 pl13 ph1):f1
  (p30*0.25 pl13 ph0):f1
  (p30*0.25 pl13 ph1):f1
 ;water suppression
```

```
 (p7 pl7 ph6):f2

 (p17 pl20 ph7):f2 (p17:sp10 ph17):f1

 1u cpds2:f2
 go=2 ph31
 1m do:f2

 10m mc #0 to 2

 F1EA(calph(ph25, +180),caldel(d0, +in0) & calph(ph15, +180) & calph(ph31, +180))  ;13C
 F2PH(calph(ph5, +90), caldel(d10, +in10)) ;15N

HaltAcqu, 1m      ;jump address for protection files
exit              ;quit

ph0  = 0
ph1  = 1
ph3  = 1
ph15 = 2
ph16 = 0 0 2 2
ph5 =  1
ph6 =  3
ph2 = 0
ph4= 1
ph25= 3
ph10= 0 3
ph12= 0 1
ph21= 0
ph22= 2

ph8= 0 0 0 0 1 1 1 1
ph7  = 2 2 2 2 0 0 0 0
ph17 = 0

ph31 = 1 3 3 1 3 1 1 3
```

REFERENCES

1. Barbara, T. M. & Williams, E. H. Modulated sequences for cross polarization during high-speed MAS. *J. Magn. Reson. 1969* **99**, 439–442 (1992).
2. Hediger, S., Meier, B. H. & Ernst, R. R. Cross polarization under fast magic angle sample spinning using amplitude-modulated spin-lock sequences. *Chem. Phys. Lett.* **213**, 627–635 (1993).
3. Gullion, T., Baker, D. B. & Conradi, M. S. New, compensated Carr-Purcell sequences. *J. Magn. Reson. 1969* **89**, 479–484 (1990).
4. Nielsen, N. C., Bildso/e, H., Jakobsen, H. J. & Levitt, M. H. Double-quantum homonuclear rotary resonance: Efficient dipolar recovery in magic-angle spinning nuclear magnetic resonance. *J. Chem. Phys.* **101**, 1805–1812 (1994).
5. Nimerovsky, E., Varkey, A. C., Kim, M., Becker, S. & Andreas, L. B. Simplified Preservation of Equivalent Pathways Spectroscopy. *JACS Au* **3**, 2763–2771 (2023).
6. Metz, G., Wu, X. L. & Smith, S. O. Ramped-Amplitude Cross Polarization in Magic-Angle-Spinning NMR. *J. Magn. Reson. A* **110**, 219–227 (1994).
7. Blahut, J., Brandl, M. J., Sarkar, R., Reif, B. & Tošner, Z. Optimal control derived sensitivity-enhanced CA-CO mixing sequences for MAS solid-state NMR – Applications in sequential protein backbone assignments. *J. Magn. Reson. Open* **16–17**, 100122 (2023).
8. Cavanagh, J. & Rance, M. Sensitivity improvement in isotropic mixing (TOCSY) experiments. *J. Magn. Reson. 1969* **88**, 72–85 (1990).
9. Westfeld, T., Verel, R., Ernst, M., Böckmann, A. & Meier, B. H. Properties of the DREAM scheme and its optimization for application to proteins. *J. Biomol. NMR* **53**, 103–112 (2012).
10. Verel, R., Baldus, M., Ernst, M. & Meier, B. H. A homonuclear spin-pair filter for solid-state NMR based on adiabatic-passage techniques. *Chem. Phys. Lett.* **287**, 421–428 (1998).
11. Verel, R., Ernst, M. & Meier, B. H. Adiabatic Dipolar Recoupling in Solid-State NMR: The DREAM Scheme. *J. Magn. Reson.* **150**, 81–99 (2001).

12. Paluch, P. *et al.* NMR Assignment of Methyl Groups in Immobilized Proteins Using Multiple-Bond 13C Homonuclear Transfers, Proton Detection, and Very Fast MAS. *Front. Mol. Biosci.* **9**, (2022).
13. Nimerovsky, E. & Goldbourt, A. Insights into the spin dynamics of a large anisotropy spin subjected to long-pulse irradiation under a modified REDOR experiment. *J. Magn. Reson.* **225**, 130–141 (2012).
14. Schnell, J. R. & Chou, J. J. Structure and mechanism of the M2 proton channel of influenza A virus. *Nature* **451**, 591–595 (2008).
15. Andreas, L. B., Eddy, M. T., Pielak, R. M., Chou, J. & Griffin, R. G. Magic Angle Spinning NMR Investigation of Influenza A M218−60: Support for an Allosteric Mechanism of Inhibition. *J. Am. Chem. Soc.* **132**, 10958–10960 (2010).
16. Shi, C. *et al.* Structure and Dynamics of the Rhomboid Protease GlpG in Liposomes Studied by Solid-State NMR. *J. Am. Chem. Soc.* **141**, 17314–17321 (2019).
17. Bligh, E. G. & Dyer, W. J. A rapid method of total lipid extraction and purification. *Can. J. Biochem. Physiol.* **37**, 911–917 (1959).
18. Bell, D. *et al.* Sedimentation of large, soluble proteins up to 140 kDa for 1H-detected MAS NMR and 13C DNP NMR – practical aspects. Preprint at https://doi.org/10.21203/rs.3.rs-3972885/v1 (2024).
19. Palmer, A. G., Cavanagh, J., Wright, P. E. & Rance, M. Sensitivity improvement in proton-detected two-dimensional heteronuclear correlation NMR spectroscopy. *J. Magn. Reson. 1969* **93**, 151–170 (1991).
20. Marion, D. & Wüthrich, K. Application of phase sensitive two-dimensional correlated spectroscopy (COSY) for measurements of 1H-1H spin-spin coupling constants in proteins. *Biochem. Biophys. Res. Commun.* **113**, 967–974 (1983).
21. Barbet-Massin, E. *et al.* Rapid Proton-Detected NMR Assignment for Proteins with Fast Magic Angle Spinning. *J. Am. Chem. Soc.* **136**, 12489–12497 (2014).

22. Thakur, R. S., Kurur, N. D. & Madhu, P. K. Swept-frequency two-pulse phase modulation for heteronuclear dipolar decoupling in solid-state NMR. *Chem. Phys. Lett.* **426**, 459–463 (2006).

23. Li, Y., Wylie, B. J. & Rienstra, C. M. Selective refocusing pulses in magic-angle spinning NMR: Characterization and applications to multi-dimensional protein spectroscopy. *J. Magn. Reson.* **179**, 206–216 (2006).

24. Shaka, A. J., Keeler, J., Frenkiel, T. & Freeman, R. An improved sequence for broadband decoupling: WALTZ-16. *J. Magn. Reson. 1969* **52**, 335–338 (1983).

25. Zhou, D. H. & Rienstra, C. M. High-performance solvent suppression for proton detected solid-state NMR. *J. Magn. Reson.* **192**, 167–172 (2008).

26. Najbauer, E. E. & Andreas, L. B. Correcting for magnetic field drift in magic-angle spinning NMR datasets. *J. Magn. Reson.* **305**, 1–4 (2019).

27. Wong, L. E., Maier, J., Wienands, J., Becker, S. & Griesinger, C. Sensitivity-Enhanced Four-Dimensional Amide–Amide Correlation NMR Experiments for Sequential Assignment of Proline-Rich Disordered Proteins. *J. Am. Chem. Soc.* **140**, 3518–3522 (2018).
